# Spatio-temporal dynamics of a planktonic system and chlorophyll distribution in a 2D spatial domain: matching model and data

**DOI:** 10.1038/s41598-017-00112-z

**Published:** 2017-03-16

**Authors:** Davide Valenti, Giovanni Denaro, Rosalia Ferreri, Simona Genovese, Salvatore Aronica, Salvatore Mazzola, Angelo Bonanno, Gualtiero Basilone, Bernardo Spagnolo

**Affiliations:** 1 Dipartimento di Fisica e Chimica, Università di Palermo, Group of Interdisciplinary Theoretical Physics and CNISM, Unità di Palermo, Viale delle Scienze, Ed. 18, I-90128 Palermo, Italy; 2 Istituto per l’Ambiente Marino Costiero, CNR, U.O.S. di Capo Granitola, Via del Faro 3, I-91020 Campobello di Mazara, TP Italy; 3 Istituto Nazionale di Fisica Nucleare, Sezione di Catania, Italy

## Abstract

Field data on chlorophyll distribution are investigated in a two-dimensional spatial domain of the Mediterranean Sea by using for phytoplankton abundances an advection-diffusion-reaction model, which includes real values for physical and biological variables. The study exploits indeed hydrological and nutrients data acquired *in situ*, and includes intraspecific competition for limiting factors, i.e. light intensity and phosphate concentration. As a result, the model allows to analyze how both the velocity field of marine currents and the two components of turbulent diffusivity affect the spatial distributions of phytoplankton abundances in the Modified Atlantic Water, the upper layer of the water column of the Mediterranean Sea. Specifically, the spatio-temporal dynamics of four phytoplankton populations, responsible for about 80% of the total *chlorophyll a*, are reproduced. Results for phytoplankton abundances obtained by the model are converted in *chlorophyll a* concentrations and compared with field data collected in twelve marine sites along the Cape Passero (Sicily)- Misurata (Libya) transect. Statistical checks indicate a good agreement between theoretical and experimental distributions of chlorophyll concentration. The study can be extended to predict the spatio-temporal behaviour of the primary production, and to prevent the consequent decline of some fish species in the Mediterranean Sea.

## Introduction

During the last decades, the study of the spatio-temporal behaviour of phytoplankton abundance assumed a role of fundamental importance to predict the effects induced by physical and hydrological changes on the fish abundances in marine ecosystems^1–5^. In particular, field observations focused on the spatial distribution of chlorophyll concentration, which is the main marker of the phytoplankton populations^2, 4–9^. These represent the base of the marine food web, and are used to estimate the biomass primary production in all aquatic ecosystems^4, 10^.

Theoretical models for population dynamics allowed to reproduce the spatio-temporal distributions of phytoplankton groups in a one-dimensional spatial domain by considering the effects of heterogeneity of the limiting factors along the water column^4, 5, 11–19^. On the other side, some authors introduced two- and three-dimensional models^20, 21^, in which the habitat of planktonic groups is considered homogenous for the nutrient and the environmental parameters are fixed constant in the whole ecosystem. In particular, these assumptions are connected with the lack of experimental data, whose availability is crucial for the estimation of some environmental variables^16, 18^, such as the light intensity, the velocity field of marine currents, the horizontal and vertical turbulent diffusivities. Moreover, unlike some one-dimensional models^4, 5, 15–18^, the numerical results for phytoplankton abundances are not converted in chlorophyll concentration, and therefore the comparison between theoretical results and experimental data can not be performed. For these reasons, the theoretical chlorophyll concentration obtained by the phytoplankton abundances has been never reproduced in the vertical water plane of the marine ecosystems. In general, the analyses are usually carried out on the horizontal water plane by using the remote sensing data^22, 23^, while it would be more useful to reproduce the field data collected in the vertical water plane, where the biodiversity of marine species can be investigated.

Here we introduce a more realistic two-dimensional model, which takes into account the physical and biochemical processes responsible for a strong spatial heterogeneity, typical of the Mediterranean Sea. This allows to reproduce the experimental spatial distributions of *chlorophyll a* (*chl a*) and *divinil chlorophyll a* (*Dvchl a*) concentration in the Strait of Sicily.

**Figure 1:**
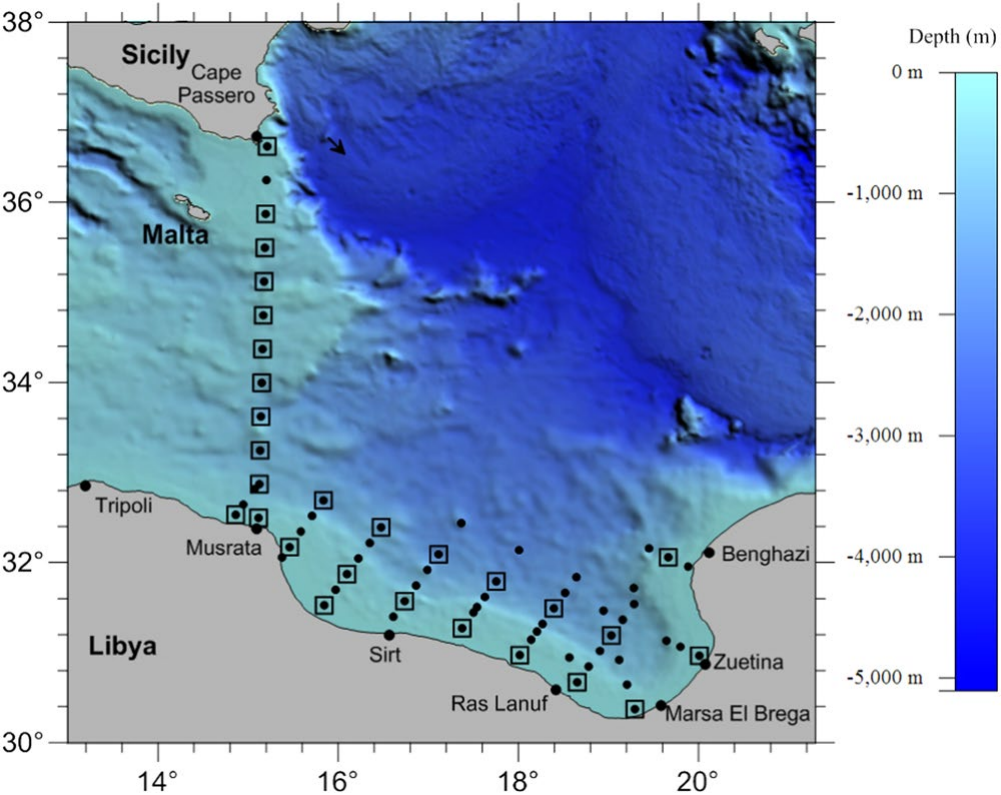
Location of the area investigated during the MedSudMed-08 cruise. The CTD stations are indicated by black circles and nutrient stations by empty squares. The bathymetry dataset was downloaded from the NOAA web portal (http://www.ngdc.noaa.gov/). Map was obtained by Surfer®^12^ from Golden Software, LLC (www.goldensoftware.com).

The sampling was carried out on a grid of 72 hydrographic profiles covering the Gulf of Syrte and the Strait of Sicily. In particular, our analysis focuses on the north-south transect (12 sites) crossing the eastern sill of the Strait of Sicily between Cape Passero and Misurata (see Fig. 1), characterized by the passage of superficial water masses in transit between the eastern and western basin of the Mediterranean Sea^24, 25^. The bathymetry dataset used in Fig. 1 was downloaded from the NOAA web portal (http://www.ngdc.noaa.gov/)^26^.

This area is interesting from a hydrological point of view since the surface marine currents of Atlantic origin, i.e. Atlantic Ionian Stream (AIS) and Atlantic Libyan Current (ALC), cause different environmental conditions for the phytoplankton growth between the Sicilian coast and Libyan coast. Moreover, the strong winds support the upwelling of nutrients close to the Sicilian coast. This favours the growth of phytoplankton populations also during the summer season when, on the contrary, it should be expected a decrease of the primary production of biomass due to the reduced water mixing. For these features, the whole area represents an important region for the spawning and growth of sardines and anchovies^7, 27^, and therefore for the fishery activities carried out in the Strait of Sicily^25^.

In this work, a two-dimensional advection-reaction-diffusion model is used to reproduce, on a vertical “water plane”, the spatio-temporal dynamics of four picophytoplankton populations. Specifically we consider the vertical “water plane” defined by the transect direction (x-axis) and the water surface-seabed direction (z-axis). The differential equations for the phytoplankton dynamics take into account the following contributions: (i) biomass production (birth and death); (ii) passive movement connected with turbulence and local transport; (iii) active movement of microorganisms along the water column.

The biomass primary production is described by a reaction term, which takes into account the nonlinear interactions between the growth of phytoplankton abundance and the two limiting factors^11, 12, 28, 29^, i.e. light intensity and nutrient concentration. Specifically, the decrease of the light intensity as a function of depth, associated with an opposite gradient for nutrients, allows each phytoplankton population to have a positive net growth rate within its production layer^4, 5, 18^. Accordingly, the magnitude and the position of the chlorophyll peaks within the two-dimensional spatial domain are strictly connected with the spatial distribution of the limiting factors^4, 30^, which however depend on the hydrological conditions of the transect investigated.

Regarding this point, it is worth to recall that the effects of mixing and local transport (passive movement) on the phytoplankton dynamics are taken into account by inserting in our equations diffusion and advection terms, respectively. Both terms are also included in the differential equation for the nutrient dynamics in order to well reproduce the two-dimensional distribution of phosphate concentration along the transect. By this way, in our analysis we consider both the direct effects of the hydrological and physical variables on the spatio-temporal behaviour of all phytoplankton groups, and those indirectly induced by the non-linear interactions between phytoplankton and the nutrient dynamics. These effects on the phytoplankton dynamics have been investigated previously in horizontal water planes^22^. However, the role of hydrological and physical variables has never been studied in a vertical water plane, where vertical chlorophyll profiles, and eventual deep chlorophyll maxima (DCMs), can be observed and analyzed. Moreover, our study presents three further novelties compared to previous investigations based on advection-diffusion-reaction models: (i) the two horizontal components of the velocity field are those measured in the transect during the oceanographic survey (15–30 July 2008); (ii) the vertical turbulent diffusivity is estimated on the basis of the Pacanowski and Philander model^31, 32^, by using the experimental data collected *in situ*; (iii) the horizontal turbulent diffusivity is fixed according to the theoretical results presented in previous works^31, 33^.

Finally, the active movement of each planktonic group is considered by using a taxis term, which depends on the behaviour of the net growth rate along the water column in accordance with previous studies^4, 5, 11^.

On this theoretical basis, we devise an advection-diffusion-reaction model which allows to reproduce the map of *chlorophyll a* concentration in the two-dimensional spatial domain of the Cape Passero-Misurata transect. Here, the hydrological parameters guarantee oligotrophic conditions during the summer season, with phosphates being the nutrient component which plays the role of limiting factor for the phytoplankton growth^4, 5, 16–18, 24, 34^.

In the following, first we consider four picophytoplankton populations^35–39^, i.e. Synechococcus, Prochlorococcus (HL-ecotype), Prochlorococcus (LL-ecotype) and the whole picoeukaryotes domain, which are the main groups in the Strait of Sicily (see Supplementary Information). Then we present a two-dimensional advection-reaction-diffusion model, obtaining the spatio-temporal dynamics for the phytoplankton abundances of these populations.

As a second step, we convert the phytoplankton abundances, expressed in *cells*/*m* ^3^, into *chl a* and *Dvchl a* concentrations, expressed in *μ*g/*dm* ^3^, by using the experimental cellular content measured by Vaulet and Courties, and the conversion curves obtained by Brunet *et al.* ^36, 40^.

As a third step, the vertical chlorophyll distributions extracted from the theoretical map are compared with the corresponding experimental profiles collected during MedSudMed-08 oceanographic survey (15–30 July 2008), by performing statistical checks based on the *χ* ^2^ test. Then, the results of these statistical tests are compared with those obtained by the “full” version of the model, in which all biological parameters are fixed (see Supplementary Information). Finally, a qualitative comparison between numerical results and experimental data is also performed for the average phosphate concentration and the average chlorophyll concentration in the Modified Atlantic Water (MAW).

## Results

The theoretical distributions of phytoplankton abundance for the four populations were obtained by solving numerically Eqs (1–7). In our analysis we considered half-saturation constants varying as a function of the position, *x*, along the transect, so that Eqs (1–7) form a “reduced” model^41^, i.e. a model with some free parameters respect to the full version, where all parameters are fixed.

In this work, the numerical method used, whose computer implementation consists in a C++ program, exploits an explicit finite difference scheme. More specifically, we used a technique based on the Method of Lines (MOL) approach, where space and time discretizations are considered separately. This method was useful to combine various discretizations for the diffusion, advection and taxis terms together with the non-linear reaction term. Indeed, we solved the differential equations of the model by performing a centered-in-space differencing for the diffusion terms, and a third-order upwind-biased differencing for the advection and taxis terms. The increment of the spatial variables is set at 5.0 km for the *x*-direction and 2.0 m for the *z*-direction, while the time step is fixed at 0.05 h. In particular, these values are chosen such as to get stability conditions for the numerical method used. On the other side, the stability analysis, performed according to previous works^20, 42–44^, indicates that the convergence of our algorithm is guaranteed^20, 42, 44^.

As initial conditions, we assumed for each picophytoplankton group a low cell concentration uniformly distributed within the domain studied in agreement with Ryabov *et al.* ^45^, while the phosphate concentrations are fixed equal to those measured along the water column in the sampling sites.

In order to obtain the steady spatial distributions, we solved numerically our equations over a time interval (*t*_*max*_ = 2 · 10^4^ *h*) long enough to reach the stationary solution^4, 18, 46^.

**Figure 2:**
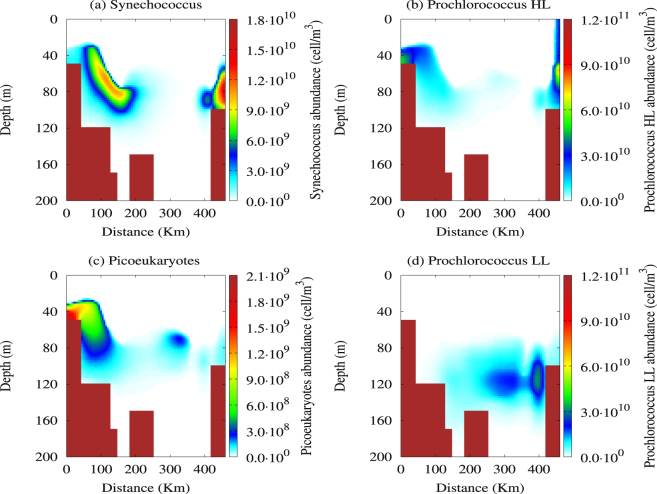
Two-dimensional distribution of the four picophytoplankton populations obtained by the reduced model. The contour maps show the cell concentrations of (**a**) Synechococcus, (**b**) Prochlorococcus HL concentrations, (**c**) picoeukaryotes and (**d**) Prochlorococcus LL at the steady state. The values of the parameters used in the reduced model are those shown in Table 1 of Supplementary Information.

In Fig. 2 we show the four picophytoplankton abundance distributions obtained by the reduced model within the two-dimensional domain. Here, it is possible to observe the presence of the abundance peak for Synechococcus and Prochlorococcus HL in shallower and intermediate layers of MAW, in correspondence of the Sicilian–Maltese shelf and the Libyan coast. Conversely, the Prochlorococcus LL abundance is very low in both the coastal zones, while an abundance peak of picoeukaryotes is mainly observed in intermediate layers of the MAW between Cape Passero and Malta. Moreover, the theoretical results indicate that the Prochlorococcus LL dominate the marine ecosystem between the Medina Sill and the Libyan coast, showing their abundance peak in deeper layers of the MAW, where the other groups are absent.

In general, from a quantitative point of view, these findings are in agreement with experimental data collected in the Strait of Sicily during the last years^24, 35, 36^. Specifically, the analysis of the numerical results shows that the average concentrations and the abundance peaks of all phytoplankton groups studied are in very good agreement with the experimental findings obtained in the Sicilian - Maltese shelf by Brunet *et al.* ^35, 36^ during a previous oceanographic survey.

According to previous studies^4, 16, 18^, to compare the theoretical results with the experimental findings, the phytoplankton abundances obtained by the model are converted into *chl a* and *Dvchl a* concentrations, setting the cellular content of Synechococcus equal to 1.18 fg *chl a* cell^−1^ ^40^, and using the curves of mean vertical profile for the other groups^35, 36^. Furthermore, we add these converted theoretical results with the contribute of chlorophyll concentration, Δ*b*_(*Dv*)*chla*_, due to the presence of nano- and micro-phytoplankton (>3 *μm*), which accounts for 20% of the total quantity of *chl a* and *Dvchl a*. This contribution is uniformly distributed along the MAW (*z*-axis), but changes along the Cape Passero–Misurata transect (*x*-axis) (see Fig. 1), taking on different values in the 12 different marine sites analyzed. We reproduce therefore the horizontal distribution of the chlorophyll contribute Δ*b*_(*Dv*)*chla*_(*x*) by calculating the 20% of fluorescence data acquired in each hydrological station, and interpolating the results obtained for all sampling sites of the transect. On the basis of these assumptions, we obtain the spatio-temporal behaviour of the total *chl a* and *Dvchl a* concentration for the whole domain investigated.

**Figure 3:**
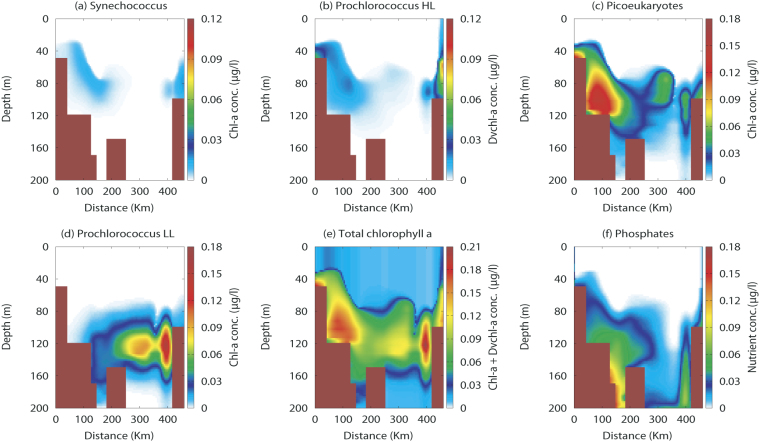
Theoretical spatial distribution of *chl a* and *Dvchl a* concentrations and phosphate concentration. The contour maps show the content of chlorophyll for (**a**) Synechococcus, (**b**) Prochlorococcus HL, (**c**) picoeukaryotes, (**d**) Prochlorococcus LL, (**e**) all phytoplankton groups, and (**f**) the phosphate concentration in the marine ecosystem investigated (Cape Passero–Misurata transect). The values of the parameters used in the reduced model are those shown in Table 1 of Supplementary Information.

The theoretical results of the *(Dv)chl a* concentrations of the four picophytoplankton groups, the total *chl a* and *Dvchl a* concentration and the phosphate concentration in stationary conditions are shown in Fig. 3. Here, we observe that the presence of high chlorophyll concentration in both the coastal zones is mainly due to Synechococcus and Prochlorococcus HL, in accordance with the theoretical results obtained for the picophytoplankton abundances (see Fig. 2). Although Synechococcus and Prochlorococcus HL prevail in the coastal zones, these two groups do not contribute significantly to the total *chl a* and *Dvchl a* concentration in the remaining part of the Cape Passero–Misurata transect. Indeed, the theoretical results indicate that the most part of chlorophyll concentration is due to the presence of picoeukaryotes and Prochlorococcus LL, which prevail in the Strait of Sicily during the summer season, when the oligotrophic conditions guarantee higher light intensity in deeper layers of the water column. In particular, these two groups dominate the marine ecosystem between the Sicilian coast and the Libyan coast in correspondence of the deep chlorophyll maximum (DCM). More specifically, the chlorophyll peak for picoeukaryotes is localized along the whole transect in the intermediate layers of the MAW, while that for Prochlorococcus LL is only obtained between the Medina Sill and the Libyan coast in deeper layers of the water column. These results are in a good agreement with the experimental findings obtained for each phytoplankton group by other authors^35–38^.

**Figure 4:**
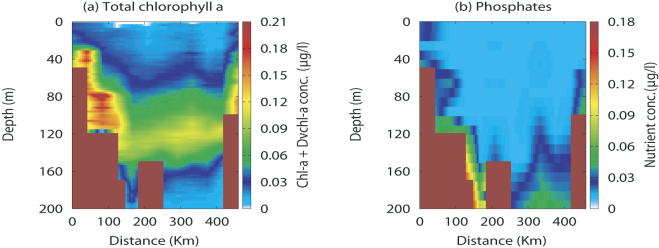
Spatial distribution of the *chlorophyll a* and phosphate concentrations obtained by the field observations. The contour maps for (**a**) the total *chl a* and *Dvchl a* concentration and (**b**) the phosphate concentration were obtained by interpolating the experimental data acquired in the twelve hydrological stations of the Cape Passero (Sicily)- Misurata (Libya) transect, during the MedSudMed-08 oceanographic survey.

**Figure 5:**
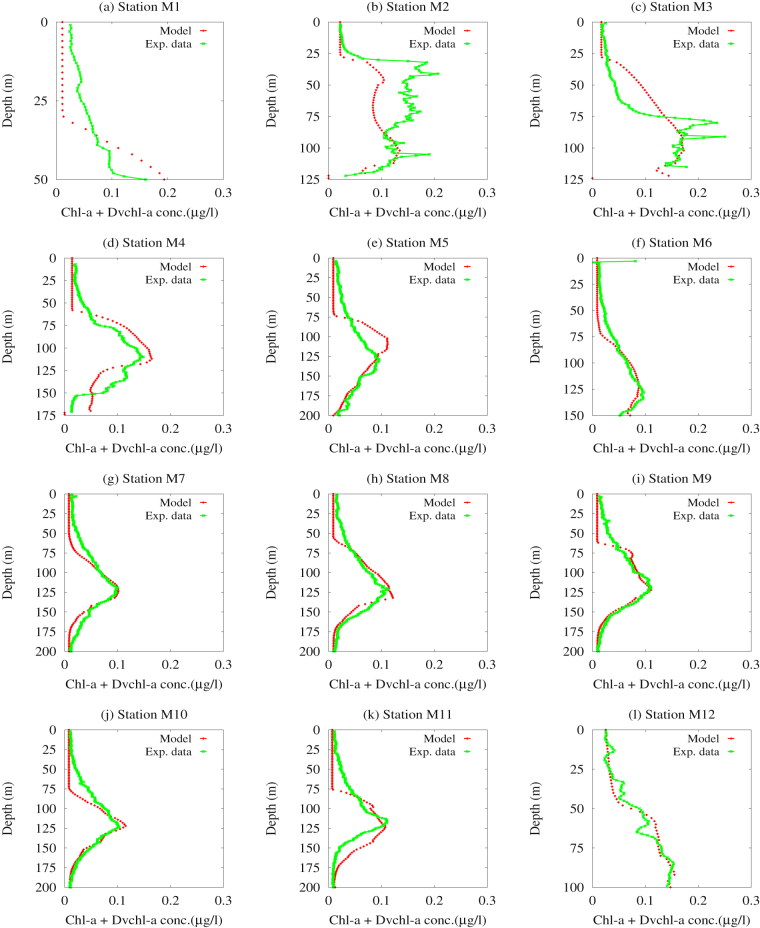
Theoretical distributions (red points) and experimental profiles (green line) of the total *chl a* and *Dvchl a* concentration. The numerical results, obtained by the reduced advection-diffusion-reaction model, are compared with the experimental data collected in the twelve hydrological stations of the Cape Passero (Sicily)- Misurata (Libya) transect, during the MedSudMed-08 oceanographic survey.

We also compare the steady spatial distribution for the total *chl a* and *Dvchl a* concentration and the phosphate concentration obtained by the model with the experimental data collected during the MedSudMed-08 oceanographic survey (see Fig. 4). From a qualitative point of view, the theoretical results for the total *chl a* and *Dvchl a* concentration are in a good agreement with experimental data in the whole transect except in a restricted area localized off the Libyan coast, and a sampling site (M2) located close to Cape Passero (see Fig. 5). These discrepancies are due to the high values taken on by the zonal component (horizontal and perpendicular to the transect) of the geostrophic currents. Our analysis, based on a two-dimensional model, does not allow indeed to include effects due to current component along the third spatial direction (*y*-axis). Finally, the comparison between the theoretical distributions and the experimental data shows a good agreement for the nutrient concentration in the whole domain, except in the hydrological stations localized close to Medina Sill. Here, in the intermediate layers of the MAW, the phosphate concentration obtained by the reduced model is overestimated respect to the values detected by the field observations. However, this behaviour can be explained by an approximative estimation of the horizontal turbulent diffusivity used within the area confined between the Sicilian coast and the Medina Sill (see the Supplementary Information).

In Fig. 5 we compare the experimental profiles of *chlorophyll a* concentration with the corresponding theoretical vertical distributions extracted from the contour maps obtained by the reduced model at the steady state. Here we observe a good qualitative agreement between experimental data (green line) and numerical results (red points) in all hydrological stations investigated. We also perform a quantitative analysis, based on the goodness-of-fit test *χ* ^2^, for the 12 stations of the transect. The results of the *χ* ^2^ test, shown in Table 2 of Supplementary Information, indicate a very good agreement between theoretical and experimental chlorophyll profiles in the hydrological stations (*M*6-*M*9) localized in the central part of the Strait of Sicily, where the geostrophic currents take on the same direction of the transect. The best reduced chi-square (${\widetilde{\chi }}_{red}^{2}=0.0021$) is observed in the station *M*12, close to the Libyan coast, where the magnitude of the geostrophic current components is very low. In other sampling sites, the results of *χ* ^2^ test indicate a worse agreement between theoretical and experimental profiles, even if the reduced chi-square ${\widetilde{\chi }}^{2}$ takes on values comparable with those obtained in previous works, where one-dimensional models were used to study the phytoplankton dynamics in summer season^4, 16^. On the whole, the two-dimensional model represents therefore an improvement compared to the corresponding one-dimensional model, because it provides a high-resolution map of the *chlorophyll a* distribution in a good agreement with the experimental findings collected in the 12 marine stations analyzed.

**Figure 6:**
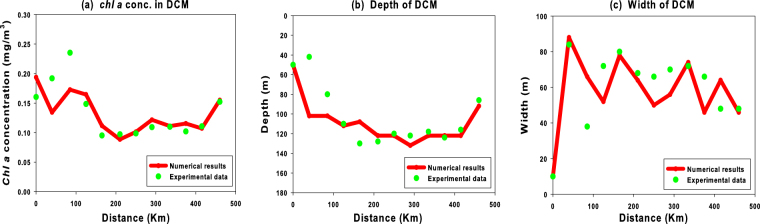
Magnitude (panel a), depth (panel b), and width (panel c) of the DCM as a function of the distance from the Cape Passero. The red lines, with a spatial resolution of 5 Km, reproduce the behaviour of the DCM obtained by the reduced model along the Cape Passero–Misurata transect. The green points describe the behaviour of the DCM obtained by the experimental data collected in the twelve hydrological stations of the transect during the sampling period (15^*th*^–30^*th*^ July 2008).

To better investigate and understand the effects of the environmental parameters on the chlorophyll distributions in the marine ecosystem studied, we analyze for the reduced model the theoretical values of the magnitude, depth and width of the DCM in all hydrological stations placed along the Cape Passero -Misurata transect. The results, shown in Fig. 6, indicate that the magnitude and depth of the DCM take on a nonmonotonic behaviour along the transect, while showing, for the most part of the stations, a clear correlation with the nutrient concentration obtained by the reduced model. In particular, we observe that the *chl a* concentration in the DCM takes on higher values in marine sites located close to the Sicilian and Lybian coasts, where larger values of the horizontal and the vertical turbulent diffusivities generate an increase of the phosphate concentration. Conversely, after a rapid decrease between Malta and the Medina Sill, the magnitude of the DCM takes on lower values in the middle of the Strait of Sicily, in accordance with the behaviour of the phosphate concentration observed in this area, characterized by lower values of the turbulent diffusivities.

Moreover, the numerical results show that, close to the coastal zones, the DCM is quite shallow because of a high phosphate concentration, while it is quite deep between the stations *M*5 and *M*11, where the oligotrophic conditions are guaranteed by lower turbulent diffusivities. On the whole, the theoretical results for the magnitude and depth of DCM are in a good agreement with the experimental ones, except in two stations (*M*2 and *M*3) located close to the Sicilian coast.

The analysis performed for the width of the DCM indicates that the experimental findings are well reproduced by the reduced model only in hydrological stations close to the coastal zones. Conversely, the width of DCM is mostly underestimated respect to the field observations in the hydrological stations localized in the middle of the Strait of Sicily. This result is connected with the difficulty to determine the correct values for the horizontal and vertical turbulent diffusivities. Finally, unlike other analysis performed on advection-diffusion-reaction models^15–18^, in this study we do not observe a connection between the magnitude and width of the DCM.

**Figure 7:**
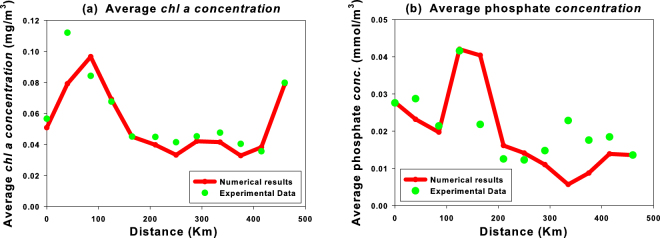
Average total *chl a* concentration and phosphate concentration as a function of the distance from the Cape Passero. Red lines show, with a spatial resolution of 5 Km, the average concentrations in the MAW obtained by the reduced model along the Cape Passero–Misurata transect. The green points are the average concentrations in the MAW obtained by the experimental data collected in correspondence of the twelve hydrological stations of the transect during the sampling period (15^*th*^–30^*th*^ July 2008).

In Fig. 7, we analyze the effects of the mixing and the local transport on the average concentration of *chlorophyll a* and phosphate within the Cape Passero -Misurata transect. Here, the theoretical results indicate that the hydrological conditions in the coastal zones generate the rising of the nutrients in the shallower layers of the MAW, causing an enhancement of the average chlorophyll concentration. In particular, an average chlorophyll peak is observed between the Sicilian coast and the Medina Sill (station *M*3), while another peak is present close to the Libyan coast (station *M*12). In the first case, the upwelling of the nutrients and the consequent growth of the phytoplankton biomass are due to the combined effect of the Atlantic Ionian Stream (AIS) and a higher water mixing. In the second case, the increase of average chlorophyll concentration is mainly due to the presence of the Atlantic Libyan Current (ALC), which carries nutrients from the Gulf of Syrte. Conversely, the reduced mixing between the Medina Sill and the Libyan coast causes in the MAW very low values of average phosphate concentration and, as a consequence, of chlorophyll concentration. In general, we observe for the average chlorophyll concentration a good agreement between the numerical results and experimental findings along the whole transect, except in the station *M*2. On the other hand, the average phosphate concentration is well reproduced close to the coastal zones, while some differences are observed in the hydrological stations localized in the middle of the transect.

In conclusion, the analysis performed in this section shows that the discrepancies between theoretical and experimental *chlorophyll a* distributions can be ascribed to: (i) the lack of experimental data on the horizontal turbulent diffusivity, necessary to better estimate the effects of high turbulence close to the coastal zones; (ii) the difficulty to determine the correct value for the vertical turbulent diffusivity in the upwelling regions; (iii) the necessity to neglect the zonal component (perpendicular to the transect) of the geostrophic velocity, because of the two-dimensional structure of the model; (iv) the lack of experimental data for the half-saturation coefficients of all picophytoplankton groups involved in our study. Moreover, the contribution of the nano- and micro-phytoplankton fraction to the total chlorophyll concentration could be underestimated close to the Sicilian coast, where the high nutrient concentration could be responsible for the growth of diatoms and Dinophytes.

Anyway, the results presented in this section indicate that the two-dimensional model provides, in wide areas of the marine ecosystem, spatial distributions of *chlorophyll a* concentration which fit quite well the field observations.

## Discussion

In this work we presented an innovative study on the spatio-temporal dynamics of phytoplankton in a marine ecosystem located in the Strait of Sicily. Initially, we performed an analysis on the two-dimensional distributions of chlorophyll and phosphate concentration obtained from data sampled, during the summer season, in twelve hydrological stations, located along the Cape Passero–Misurata transect. Here, field observations indicate the presence of a strong spatial heterogeneity in physical and chemical variables such as velocity field, water density, and nutrient concentration. Moreover, we observed that the characteristics of the vertical chlorophyll profile in each sampling site are strictly connected with the horizontal velocities of marine currents, turbulent diffusivities (vertical and horizontal), and nutrient concentration at the boundaries of the ecosystem.

In this work we used a two-dimensional advection-reaction-diffusion model, while neglecting the terms of the local transport in the direction perpendicular to the transect, since in the most hydrological stations investigated during the summer period the velocity field is parallel to the transect, and the contribution of the zonal geostrophic velocity on the local transport can be therefore neglected (see Supplementary Information and, in particular, Fig. 1).

The equations of the reduced model were solved by numerical methods, simulating the spatio-temporal dynamics of four phytoplankton populations, responsible for about 80% of the total *chlorophyll a* inside the Modified Atlantic Water, that is the upper layer of the water column of the Mediterranean Sea. Then, by using the conversion rates obtained in previous works^36, 40^ we obtained the *chl a* and *Dvchl a* theoretical concentrations, and compared them with the corresponding field data.

The spatio-temporal dynamics of the four planktonic populations was obtained by the model equations, taking into account explicitly the effects of environmental variables. More in detail, we considered the different hydrological features of the 12 stations along the transect investigated. In particular, we found that the strong stratification of the Mediterranean Sea in the summer season^2, 47, 48^ causes a reduced nutrient concentration in the shallower layers of the MAW, and therefore a low chlorophyll concentration in the most part of the Strait of Sicily (except in the coastal zones). Conversely, the strong wind at the marine surface induces the upwelling of nutrients close to the Sicilian coast, while favouring the growth of phytoplankton populations also during the summer season. Moreover, an increase of the chlorophyll concentration is also observed close to Libyan coast due to the presence of the Atlantic Libyan Current (ALC), which carries nutrients from the Gulf of Syrte.

We note also that in our equations the effects of the mixing, local transport (passive movement), and non-linear interactions between phytoplankton and nutrient dynamics are modeled by the diffusion and advection terms. Moreover, our model exploits values of hydrological and physical variables experimentally measured, including horizontal components of the velocity field and vertical turbulent diffusivity obtained from field observations.

These aspects represent a novelty within the landscape of the modeling of phytoplankton dynamics in a vertical water plane. Moreover, the model provides a tool for a better and deeper understanding of the role played by hydrological conditions on the spatial distribution of phytoplankton abundances, which represent the base of the food web for marine ecosystems. Our theoretical findings indeed could be used to analyze the role of environmental variables, through the modifications induced in the phytoplankton distributions, in the dynamics of zooplankton populations and fish species in the Strait of Sicily^7, 25, 27, 49^. Indeed previous investigations showed that the anchovy population prevails in the upwelling region^3, 49, 50^, where the high nutrient concentrations favour the growth of larger size phytoplankton groups, which are at the basis of the food chain of this fish species. On the other hand, sardines, which are able to capture smaller size phytoplankton and zooplankton, dominate geographical areas with reduced water mixing during the most part of the year^50^.

In our analysis, we considered the presence of two limiting factors responsible for the competition among the four picophytoplankton populations modeled. The biological parameters were set to values typical of these planktonic groups, according to previous field observations carried out in the Strait of Sicily^10, 29, 35, 36, 51–58^. Specifically, environmental conditions were fixed according to field observations, and biological parameters were set in such a way to guarantee, along the water column, the coexistence of all four populations^4, 5, 12, 16–18, 45^.

Moreover, the heterogeneous composition of each picophytoplankton population showed a spatial variability, modeled by varying the values of half-saturation constants along the transect analyzed. About this, our modeling actually pays the lack of an adequate knowledge of the mechanism used by phytoplankton cells to absorb nutrient molecules and energy from the marine waters under the different environmental conditions^9^. Indeed, a correct modeling of phytoplankton dynamics should also take into account that the half-saturation constants depend on the nutrient concentration and the light intensity, which change as a function of the position (*x*, *z*) within the two-dimensional domain. However, a preliminary analysis (results here not reported) based on the full model has been performed, obtaining spatial distributions of phytoplankton abundances in a quite good agreement (except in stations M1, M2, M3) with the field data acquired along the Cape Passero - Misurata transect.

Anyway, due to the characteristics of the marine ecosystem analyzed (see Supplementary Information), in view of better reproducing the experimental data, we assumed half-saturation constants as space dependent parameters. So we used a reduced model in which the values of half-saturation constants depend on the position *x* along the transect, instead of the full model where all biological parameters are fixed. In particular, the values of half-saturation constants for each station were initially fixed in such a way that the boundaries of the production layer of each population are localized at depths determined according to the theoretical expression by Ryabov *et al.* ^14, 45^. The final values of half-saturation constants were obtained by using an iterative procedure, based on *χ* ^2^ test, which optimizes the theoretical profiles of chlorophyll distributions compared with those obtained by field data (see Supplementary Information). Moreover, in our analysis we imposed an additional constraint: in all hydrological stations the theoretical abundances (cell concentrations) for each phytoplankton population have to be in a good agreement with those experimentally observed by Brunet *et al.* ^35, 36^. Our theoretical results satisfy also this condition.

In our analysis the theoretical 2D distribution of chlorophyll concentration was obtained by exploiting the conversion curves^36^. Then we extracted from the chlorophyll map the theoretical chlorophyll profiles, comparing them with the experimental profiles collected in the 12 stations during the MedSudMed-08 oceanographic survey (15–30 July 2008). At this stage we applied the above cited optimization procedure, obtaining theoretical profiles in a good agreement with those obtained from field observations, and strongly dependent on the hydrological variables (horizontal velocities and turbulent diffusivities), characterized by different values in the various stations analyzed. Moreover, from a qualitative point of view, we observed that the magnitude, depth and width of the DCMs predicted by the reduced model match quite well those obtained by field observations. We also calculated, in each station, the average chlorophyll concentration along the water column, finding a good agreement between theoretical results and experimental data in the whole transect, except in station *M*2. Finally, the good agreement between predicted results and experimental data for the average phosphate concentration represented a further validation of the reduced model proposed.

We also compared quantitatively theoretical and experimental chlorophyll profiles, performing the *χ* ^2^ goodness-of-fit test. The results showed that the best reduced chi-square is obtained for the hydrological stations localized between the Medina Sill and the Libyan coast, where the turbulent diffusivities take on low values and the geostrophic currents are parallel to the direction of the transect. Conversely, the worst values of *χ* ^2^ are obtained between the Sicilian coast and the Medina Sill, where the high vertical turbulent diffusivity triggers the upwelling of nutrient from deeper layers, and the direction of the marine currents is not parallel to the transect.

Afterwards, we also compared the results of the *χ* ^2^ test for the reduced and full models, by using the Akaike Information Criterion and the Cohen’s effect size measure^41, 59^. In particular, both statistical checks show that also the full model is able to well reproduce the experimental chlorophyll profiles in the most part of the transect, even if the best *χ* ^2^ values are obtained by the reduced model. As a final remark we note that, from a conceptual point of view it appears more correct to use the reduced model, which takes into account the spatial variability of the half-saturation constants. These parameters indeed depend strongly on the environmental conditions, such as water turbidity which influences the light intensity along the water column, and nutrient concentration. The reduced model allows to take into account this dependence by including site specific values for half-saturation constants. Finally, the small values of the Cohen’s effect size measure allows to extend to the full model the good agreement between experimental and theoretical distributions of chlorophyll, obtained when the reduced model is used.

In general, this study indicates that the advection-diffusion-reaction model proposed is able to reproduce correctly the spatial distributions of the chlorophyll concentration, when biological and environmental parameters are set to values typical (experimentally measured) of the ecosystem considered (Strait of Sicily). Anyway, it is expected that the theoretical distributions for chlorophyll concentration could be improved by using a 3D model, which should allow to take into account the effects of diffusion and advection along the third dimension (perpendicularly to the transect), here not considered because of the 2D structure of the experimental dataset. In particular, the implementation of the 3D model would allow to obtain a better agreement between the theoretical results and the experimental data in the hydrological stations where the velocity field is not parallel to the transect. Moreover, a better modeling needs a deeper knowledge of biological and environmental parameters such as: (i) horizontal and vertical turbulent diffusivities close to coastal zones; (ii) dependence of the half-saturation constants on the spatial distributions of nutrient concentration and light intensity; (iii) real contribution of the nano- and micro-phytoplankton fraction to the chlorophyll concentration in the coastal zones; (iv) the inflow and outflow of nutrients and chlorophyll coming from the boundaries of the marine ecosystem.

In conclusion, the model proposed in this work appears as a valid candidate to predict the chlorophyll spatial distribution in marine ecosystems characterized by heterogeneous environmental conditions. Specifically, our findings indicate that the model is able to reproduce phytoplankton dynamics in aquatic ecosystems with different levels of eutrophication. We note that this model can be extended, including higher trophic levels such as zooplankton populations, in view of reproducing and predicting the spatio-temporal behaviour of fish populations^5, 21^. Finally, we wish to notice how the model and, more in general, the whole approach proposed represent a powerful tool to analyze and predict the effects of global warming on phytoplankton dynamics, in view of preventing the decline of the primary production in marine ecosystems^9, 18^.

## Materials and Methods

### Environmental data

The experimental data were collected in the period 15^*th*^–30^*th*^ July 2008 during the MedSudMed-08 Oceanographic Survey carried out in the Central Mediterranean Sea (CMED) on board the CNR R/V Urania. This area was investigated by using a grid of 72 hydrographic profiles, which covered the Gulf of Syrte and a north-south transect crossing the eastern sill of the Strait of Sicily between Cape Passero and Misurata^24^(see Fig. 1). Specifically, the sampling sites were located on a grid of 12 × 12 nautical miles in the Gulf of Syrte. The distance between two consecutive sites was about 22.5 nautical miles along the Cape Passero-Misurata transect. Hydrological conditions remained constant for the entire sampling period and were representative of the oligotrophic Mediterranean Sea in summer.

For each sampling site, continuous vertical profiles of conductivity, temperature, density and pressure were collected from the surface to the bottom of the water column by means of a CTD-rosette system, consisting of a CTD SBE 911 plus probe (Sea-Bird Inc.), and a General Oceanics rosette with 24 Niskin Bottles^24, 60^. Simultaneously, the *chlorophyll a* fluorescence data (*chl a* concentration in *μ*g/l) were acquired by using the Chelsea Aqua 3 sensor mounted on the probe. All parameters were processed, generating for each site a text file, in which the experimental values are given with a 1 m step^16–18^.

The vertical profiles of the two horizontal components of the current velocity have been acquired by using a Lowered Acoustic Doppler Current Profiler system (LADCP) and computed by applying the Lamont-Doherty Earth Observatory (LDEO) processing software^61^. At the same time, the main surface circulation patterns were evaluated by the Absolute Dynamic Topography during the investigated period.

The horizontal velocities were collected in all sampling sites of the transect except in the station *M*12. Here, we assumed that the velocities were the same as in the nearest station *L*2718. Indeed, we recall that the vertical profiles of horizontal marine currents and water density were necessary to estimate the spatial distribution of the vertical turbulent diffusivity^31, 32^ along the whole Cape Passero-Misurata transect (see Supplementary Information).

The seawater samples to perform the dissolved inorganic nutrients measurements were collected during the oceanographic survey at standard depths in all hydrological stations of the transect investigated except in the M2 site. The nutrient samples were stored at −20 °*C* without carrying out any preliminary filtration. Nitrite, nitrate, silicate and phosphate concentrations were measured successively in laboratory by using a Brän-Luebbe AutoAnalyzer, and following classical methods with slight modifications^24, 62^.

### The model

The dynamics of the primary production of biomass is studied by using an advection-diffusion-reaction model^4, 11, 12, 45^. In particular, we investigate the spatio-temporal behaviour of four picophytoplankton populations, which interact with each other indirectly through the competition for light intensity and nutrient concentration. By solving the equations of the model, we obtain the steady spatial distribution of cell concentration for the populations analyzed in the Strait of Sicily, i.e. Synechococcus, Prochlorococcus HL, picoeukaryotes, and Prochlorococcus LL, indicated by *b*_1_(*x*, *z*, *t*), *b*_2_(*x*, *z*, *t*), *b*_3_(*x*, *z*, *t*), and *b*_4_(*x*, *z*, *t*), respectively. Here, *b*_*i*_(*x*, *z*, *t*) denotes the phytoplankton abundance (cells per unit volume) of *i-th* population in the position (*x*, *z*) within the two-dimensional domain at time t. Moreover, the spatial distributions of the phosphate concentration *R*(*x*, *z*, *t*) and light intensity *I*(*x*, *z*, *t*) are obtained. In our study, the domain is composed by the sum of seven rectangular subdomains, which reproduce the bathymetric profile of the transect together with the vertical boundaries localized in the two hydrological stations close to the two coasts. Specifically, *z* represents the depth of the water column from the surface (*z* = 0) to the bottom (*z* = *z*_*b*_) of each subdomain, while *x* indicates the distance from the Sicilian coast, measured from Cape Passero (*x* = 0) to Misurata (*x*_*l*_ = 0) along the transect.

The dynamics of the picophytoplankton abundance is modeled by including three processes^11, 12, 20^: (i) net growth (reaction term); (ii) active/passive movement (taxis/advection term); (iii) movement due to turbulence (diffusion term).

The reaction term describes how the limiting resources affect the net phytoplankton growth rate (*G*_*i*_(*x*, *z*, *t*))^11, 14, 28, 45, 63, 64^, which depends on the balance between the gross production rate per capita and the mortality. In particular, the gross production rates are given by ${\rm{\min }}\,\{{f}_{{I}_{i}}(I),{f}_{{R}_{i}}(R)\}$, where ${f}_{{I}_{i}}(I)$ and ${f}_{{R}_{i}}(R)$ are obtained by the Michaelis-Menten formulas for light intensity and phosphate concentration^4, 16, 18, 19^, while the mortality due to respiration, death, and grazing is described by the specific loss rates (*m*_*i*_).

The taxis term provides a realistic description of motility skills of the planktonic groups, and depends on the swimming velocity *v*_*i*_ of each population, which is a function of the gradient of the net growth rate (∂*G*_*i*_(*x*, *z*, *t*)/∂*z*)^11^. In particular, this function is defined as ${v}_{i}=+{v}_{i}^{s}$(sinking phytoplankton) if ∂*G*_*i*_(*x*, *z*, *t*)/∂*z* > 0, ${v}_{i}=-{v}_{i}^{s}$ (buoyant phytoplankton) if ∂*G*_*i*_(*x*, *z*, *t*)/∂*z* < 0, and *v*_*i*_ = 0 (motionless phytoplankton) if ∂*G*_*i*_(*x*, *z*, *t*)/∂*z* = 0, where ${v}_{i}^{s}$ is a constant parameter, whose value (positive) is calculated for each population according to Raven^51^.

The non-active movement of all phytoplankton groups depends on the turbulence (diffusion term) and the local transport (advection term) of biomass^4, 20, 45^. In particular, the diffusion term reproduces the effects of the turbulence on the two-dimensional distribution of phytoplankton groups through the horizontal (*D*_*h*_(*x*)) and the vertical (*D*_*v*_(*x*, *z*)) turbulent diffusivities, which change as a function of the depth (*z*) and the length (*x*), while remaining constant with the time. Hence, as a preliminary step, we obtain the values of vertical turbulent diffusivity from field observations by exploiting the model devised by Pacanowski and Philander^31^. The horizontal turbulent diffusivity is fixed in accordance with values estimated by other authors^31, 33, 65^ (see Supplementary Information).

The advection term allows to describe the effects on the picophytoplankton distributions induced by the horizontal velocity component (*v*_*h*_(*x*, *z*)) of the marine currents along the *x*-direction. Since in the most part of the transect the velocity field is parallel to the *x*-direction (see Supplementary Information and, in particular, Fig 1), we do not parameterize the local transport of nutrients and chlorophyll along the *y*-direction due to the zonal geostrophic velocity. The horizontal velocities (constant in time) are calculated by using the experimental data collected during the oceanographic MedSudMed-08 survey (see Supplementary Information).

The phosphate concentration *R*(*x*, *z*, *t*) is further increased by the recycling process of the dead phytoplankton. Moreover, the effects of the local transport and turbulence, responsible for the mixing of nutrients in the 2D domain, are taken into account by inserting in the differential equation for the phosphate concentration an advection term and two diffusion terms, respectively.

Finally, the light intensity *I*(*x*, *z*, *t*) is assumed to depend on the site considered along the transect, and to decrease exponentially with the depth *z*, according to the Lambert-Beer’s law^4, 29, 66, 67^.

Thus, the four-population model is defined by the following equations:

1 $$\begin{array}{rcl}\frac{\partial {b}_{i}(x,z,t)}{\partial t} & = & {b}_{i}(min({f}_{{I}_{i}}(I),{f}_{{R}_{i}}(R))-{m}_{i})+\frac{\partial }{\partial z}[{D}_{v}(x,z)\frac{\partial {b}_{i}(x,z,t)}{\partial z}]\\  &  & +\frac{\partial }{\partial x}[{D}_{h}(x)\frac{\partial {b}_{i}(x,z,t)}{\partial x}]-\frac{\partial }{\partial x}[{v}_{h}(x,z){b}_{i}(x,z,t)]\\  &  & -\frac{\partial }{\partial z}[{v}_{i}(x,z,t){b}_{i}(x,z,t)]\end{array}$$

2 $$\begin{array}{rcl}\frac{\partial R(x,z,t)}{\partial t} & = & -\sum \frac{{b}_{i}(x,z,t)}{{Y}_{i}}\cdot min({f}_{{I}_{i}}(I),{f}_{{R}_{i}}(R))+\frac{\partial }{\partial z}[{D}_{v}(x,z)\frac{\partial R(x,z,t)}{\partial z}]\\  &  & +\frac{\partial }{\partial x}[{D}_{h}(x)\frac{\partial R(x,z,t)}{\partial x}]-\frac{\partial }{\partial x}[{v}_{h}(x,z)R(x,z,t)]\\  &  & +\sum {\varepsilon }_{i}{m}_{i}\frac{{b}_{i}(x,z,t)}{{Y}_{i}}\end{array}$$

3 $$I(x,z,t)={I}_{in}(x)exp\{-{\int }_{0}^{z}[\sum {a}_{i}\cdot chl\,{a}_{i}(x,Z,t)+{a}_{bg}]dZ\},$$

with i = 1, ..., 4. Here, *ε*_*i*_, *m*_*i*_, 1/*Y*_*i*_, and *a*_*i*_ are the nutrient recycling coefficient, the specific loss rate, the nutrient content, and the *chl a*-normalized average absorption coefficient of the *i-th* picophytoplankton population, respectively; *a*_*bg*_ is the background turbidity; *I*_*in*_(*x*) is the incident light intensity at the water surface, taking on different values along the transect (*x*-direction).

Respect to the phytoplankton populations the marine ecosystem is closed^20^, that is we assume that no biomass can enter or leave the area investigated. Therefore, we fix the biomass fluxes to vanish at the boundaries of the domain:

4 $${[{D}_{v}(x,0)\frac{{\rm{\partial }}{b}_{i}}{{\rm{\partial }}z}-{v}_{i}{b}_{i}]|}_{z=0}=0,\qquad {[{D}_{v}(x,{z}_{b})\frac{{\rm{\partial }}{b}_{i}}{{\rm{\partial }}z}-{v}_{i}{b}_{i}]|}_{z={z}_{b}}=0,$$

5 $${[{D}_{h}(0)\frac{{\rm{\partial }}{b}_{i}}{{\rm{\partial }}x}-{v}_{h}{b}_{i}]|}_{x=0}=0,\qquad {[{D}_{h}({x}_{l})\frac{{\rm{\partial }}{b}_{i}}{{\rm{\partial }}x}-{v}_{h}{b}_{i}]|}_{x={x}_{l}}=0.$$

For the nutrient (phospates) we assume: (i) no nutrient flux through the water surface; (ii) nutrient concentration at the other three boundaries fixed equal to the values measured during the oceanographic survey. Therefore, we set the phosphate fluxes at the boundaries of the domain as follows:

6 $${\frac{{\rm{\partial }}R}{{\rm{\partial }}z}|}_{z=0}=0,\qquad R(x,{z}_{b})={R}_{in}(x,{z}_{b}).$$

7 $$R(0,z)={R}_{in}(0,z),\qquad R({x}_{l},z)={R}_{in}({x}_{l},z).$$

Equations (1–7) represent the two-dimensional advection-diffusion-reaction model used to describe and reproduce the spatio-temporal dynamics of phytoplankton abundance.

## Electronic supplementary material

Supplementary Information

## References

[1] Bopp L (2001). Potential impact of climate change on marine export production. Global. Biogeochem. Cy.

[2] Patti B (2010). Role of physical forcings and nutrient availability on the control of satellite-based chlorophyll *a* concentration in the coastal upwelling area of the Sicilian Channel. Sci. Mar..

[3] Bonanno A (2013). Influence of environmental variability on anchovy early life stages (*Engraulis encrasicolus*) in two different areas of the Central Mediterranean Sea. Hydrobiologia.

[4] Valenti D, Denaro G, Spagnolo B, Conversano F, Brunet C (2015). How diffusivity, thermocline and incident light intensity modulate the dynamics of deep chlorphyll maximum in Tyrrhenian Sea. PLoS ONE.

[5] Valenti D (2016). The role of noise on the steady state distributions of phytoplankton populations. J. Stat. Mech.

[6] Jennings S, Kaiser MJ, Reynolds JD (2001). Marine Fisheries Ecology.

[7] Cuttitta A (2003). Anchovy egg and larval distribution in relation to biological and physical oceanography in the Strait of Sicily. Hydrobiologia.

[8] Weston K, Fernand L, Mills DK, Delahunty R, Brown J (2005). Primary production in the deep chlorophyll maximum of the central North Sea. J. Plankton Res..

[9] Kiørboe, T. *A mechanistic approach to plankton ecology* (Princeton University Press, 2008).

[10] Veldhuis MJW, Timmermans KR, Croot P, Van Der Wagt B (2005). Picophytoplankton; a comparative study of their biochemical composition and photosynthetic properties. J. Sea Res..

[11] Klausmeier CA, Litchman E (2001). Algal games: the vertical distribution of phytoplankton in poorly mixed water columns. Limnol. Oceanogr..

[12] Huisman J, Thi NNP, Karl DM, Sommeijer B (2006). Reduced mixing generates oscillations and chaos in the oceanic deep chlorophyll maximum. Nature.

[13] Ryabov AB, Blausius B (2008). Population growth and persistence in a heterogeneous environment: the role of diffusion and advection. Math. Model. Nat. Phenom.

[14] Ryabov A (2012). Phytoplankton competition in deep biomass maximum. Theor. Ecol.

[15] Valenti D (2012). Picophytoplankton dynamics in noisy marine environment. Acta Phys. Pol. B.

[16] Denaro G (2013). Spatio-temporal behaviour of the deep chlorophyll maximum in Mediterranean Sea: Development of a stochastic model for picophytoplankton dynamics. Ecol. Complex..

[17] Denaro G (2013). Stochastic dynamics of two picophytoplankton populations in a real marine ecosystem. journalActa Phys. Pol. B.

[18] Denaro G (2013). Dynamics of two picophytoplankton groups in Mediterranean Sea: Analysis of the deep chlorophyll maximum by a stochastic advection-reaction-diffusion model. PLoS ONE.

[19] Valenti D (2016). Stochastic models for phytoplankton dynamics in Mediterranean Sea. Ecol. Complex..

[20] Thi NNP, Huisman J, Sommeijer BP (2005). Simulation of three-dimensional phytoplankton dynamics: competition in light-limited environments. J. Comput. Appl. Math..

[21] Liu QX, Jin Z, Li BL (2008). Resonance and frequency-locking phenomena in spatially extended phytoplankton-zooplankton system with additive noise and periodic forces. J. Stat. Mech.-Theory Exp.

[22] Hernández-Carrasco I, Rossi V, Hernández-Garca E, Garçon V, López C (2014). The reduction of plankton biomass induced by mesoscale stirring: A modelling study in the Benguela upwelling. Deep-Sea Res. I.

[23] Falcini F (2015). The Role of Hydrodynamics Processes on Anchovy Eggs and Larvae Distribution in the Sicily Channel (Mediterranean Sea): A Case Study for the 2004 Data Set. PLoS ONE.

[24] Placenti F (2013). Water masses and nutrient distribution in the Gulf of Syrte and between Sicily and Libya. J. Marine Syst..

[25] Rinaldi E, Buongiorno Nardelli B, Volpe G, Santoleri R (2014). Chlorophyll distribution and variability in the Sicily Channel (Mediterranean Sea)as seen by remote sensing data. Cont. Shelf Res..

[26] Amante, C., Eakins, B. W. ETOPO1 1 Arc-Minute Global Relief Model: Procedures, Data Sources and Analysis. NOAA Technical Memorandum NESDIS NGDC-24 (National Geophysical Data Center NOAA, USA, 2009).

[27] Agostini VN, Bakun A (2002). ‘Ocean triads’ in the Mediterranean Sea: physical mechanisms potentially structuring reproductive habitat suitability (with example application to European anchovy, *Engraulis encrasicolus*). Fish. Oceanogr..

[28] Klausmeier CA, Litchman E, Levin SA (2007). A model of flexible uptake of two essential resources. J. Theor. Biol..

[29] Hickman A, Dutkiewicz S, Williams R, Follows M (2010). Modelling the effects of chromatic adaptation on phytoplankton community structure in the oligotrophic ocean. Mar. Ecol. Prog. Ser..

[30] Norberg J (2004). Biodiversity and ecosystem functioning: a complex adaptive systems approach. Limnol. Oceanogr..

[31] Pacanowski RC, Philander SGH (1981). Parameterization of Vertical Mixing in Numerical Models of Tropical Oceans. J. Phys. Oceanogr..

[32] Peters H, Gregg MC, Toole JM (1988). On the parameterization of Equatorial Turbulence. J. Geophys. Res..

[33] Massel, S. R. *Fluid Mechanics for Marine Ecologists* (Springer-Verlag, Berlin Heidelberg, 1999).

[34] Ribera d’Alcalà M, Civitarese G, Conversano F, Lavezza R (2003). Nutrient ratios and fluxes hint at overlooked processes in the Mediterranean Sea. J. Geophys. Res..

[35] Brunet C, Casotti R, Vantrepotte V, Corato F, Conversano F (2006). Picophytoplankton diversity and photoacclimation in the Strait of Sicily (Mediterranean Sea) in summer. I. Mesoscale variations. Aquat. Microb. Ecol..

[36] Brunet C, Casotti R, Vantrepotte V, Conversano F (2007). Vertical variability and diel dynamics of picophytoplankton in the Strait of Sicily, Mediterranean Sea, in summer. Mar. Ecol. Prog. Ser..

[37] Garczarek L (2007). High vertical and low horizontal diversity of *Prochlorococcus* ecotypes in the Mediterranean Sea in summer. FEMS Microbiol Ecol.

[38] Mella-Flores D (2011). Is the distribution of Prochlorococcus and Synechococcus ecotypes in the Mediterranean Sea affected by global warming?. Biogeosciences.

[39] La Ferla R (2012). Vertical distribution of the prokaryotic cell size in the Mediterranean Sea. Helgol. Mar. Res..

[40] Morel A, Ahn YH, Partensky F, Vaulot D, Claustre H (1993). *Prochlorococcus* and *Synechococcus*: A comparative study of their optical properties in relation to their size and pigmentation. J. Mar. Res..

[41] Newsom JT (2015). Longitudinal Structural Equation Modeling: A Comprehensive Introduction.

[42] Roache PJ (1998). Fundamentals of Computational Fluid Dynamics.

[43] Tveito A, Winther R (1998). Introduction to Partial Differential Equations: A Computational Approach.

[44] Hundsdorfer W, Verwer JG (2003). Numerical solution of time-dependent advection-diffusion-reaction equations publisher.

[45] Ryabov AB, Rudolf L, Blasius B (2010). Vertical distribution and composition of phytoplankton under the influence of an upper mixed layer. J. Theor. Biol..

[46] Fennel K, Boss E (2003). Subsurface maxima of phytoplankton and chlorophyll: Steady-state solutions from a simple model. Limnol. Oceanogr..

[47] Behrenfeld MJ (2006). Climate-driven trends in contemporary ocean productivity. Nature.

[48] Barale V, Jaquet JM, Ndiaye M (2008). Algal blooming patterns and anomalies in the Mediterranean Sea as derived from the SeaWiFS data set (1998–2003). Remote Sens. Environ..

[49] Basilone G (2006). Effect of habitat conditions on reproduction of the European anchovy (*Engraulis encrasicolus*) in the Strait of Sicily. Fish. Oceanogr.

[50] D’Alelio D, Libralato S, Wyatt T, Ribera d’Alcalá S (2016). Ecological-network models link diversity, structure and function in the plankton food-web. Sci. Rep.

[51] Raven JA (1998). The twelfth tansley lecture. Small is beautiful: the picophytoplankton. Funct. Ecol.

[52] Raven JA, Finkel ZV, Irwin AJ (2005). Picophytoplankton: bottom-up an top-down controls on ecology and evolution. J. Geophys. Res..

[53] Dimier C, Brunet C, Geider R, Raven J (2009). Growth and photoregulation dynamics of the picoeukaryote *Pelagomonas calceolata* in fluctuating light. Limnol. Oceanogr..

[54] Thingstad TF, Sakshaugh E (1990). Control of phytoplankton growth in nutrient recycling ecosystems. Theory and terminology. Mar. Ecol. Prog. Ser..

[55] Quevedo M, Anadón R (2001). Protist control of phytoplankton growth in the subtropical north-east Atlantic. Mar. Ecol. Prog. Ser..

[56] Moore LR, Goericke R, Chisholm SW (1995). Comparative physiology of synechococcus and prochlorococcus: influence of light and temperature on growth, pigments, fluorescence and absorptive properties. Mar. Ecol. Prog. Ser..

[57] Bertilsson S, Berglund O, Karl DM, Chisholm SW (2003). Elemental composition of marine *Prochlorococcus* and *Synechococcus*: implications for the ecological stoichiometry of the sea. Limnol. Oceanogr..

[58] Timmermans KR, van der Wagt B, Veldhuis MJW, Maatman A, de Baar HJW (2005). Physiological responses of three species of marine pico-phytoplankton to ammonium, phospahte, iron and light limitation. J. Sea Res..

[59] Akaike H (1987). Factor analysis and AIC. Psychometrika.

[60] Bonanno A (2014). Variability of water mass properties in the Strait of Sicily in summer period of 1998–2013. Ocean Sci..

[61] Visbeck M (2002). Deep velocity profiling using lowered acoustic Doppler current profilers: bottom track and inverse solutions. J. Atmos. Ocean. Technol..

[62] Grasshoff, K., Kremling, K., Ehrhardt, M. *Methods of Seawater Analysis* (Wiley-Vch Verlag, Weinheim, Germany, 1999).

[63] Mei ZP, Finkel ZV, Irwin AJ (2009). Light and nutrient availability affect the size-scaling of growth in phytoplankton. J. Theor. Bio.

[64] Bougaran G, Bernard O, Sciandra A (2010). Modeling continuous cultures of microalgae colimited by nitrogen and phosphorus. J. Theor. Biol..

[65] Katz EJ, Bruce JG, Petrie BD (1979). Salt and mass flux in the Atlantic Equatorial Undercurrent. Deep-Sea Res.

[66] Shigesada N, Okubo A (1981). Effects of competition and shading in planktonic communities. J. Math. Biol..

[67] Kirk, J. T. O. *Light and Photosynthesis in Aquatic Ecosystems* (2^nd^ edition) (Cambridge University Press, 1994).

