# Supplementary material for: Spatio-temporal dynamics of a planktonic system and chlorophyll distribution in a 2D spatial domain: matching model and data
Source: Sci Rep. 2017 Mar 16;7:220. doi: 10.1038/s41598-017-00112-z (PMC5427904; doi:10.1038/s41598-017-00112-z)
Supplement: Supplementary file 1 — Supplementary Information [file 41598_2017_112_MOESM1_ESM.pdf]

SUPPLEMENTARY INFORMATION

Spatio-temporal dynamics of a planktonic system and chlorophyll distribution in a 2D spatial domain: matching model and data

Davide Valenti[a], Giovanni Denaro, Rosalia Ferreri, Simona Genovese, Salvatore Aronica, Salvatore Mazzola, Angelo Bonanno, Gualtiero Basilone, Bernardo Spagnolo

Correspondence should be addressed to D.V. (email: davide.valenti@unipa.it)

SUPPLEMENTARY METHODS

Analysis of environmental data

The vertical distributions of hydrological parameters, i.e. temperature and salinity, were used to identify the different water masses present in the studied area [1, 2]. Here, the field observations indicate the presence of the Modified Atlantic Water (MAW), i.e. the upper layer of the water column of Atlantic origin localized from the surface down to 200 m. In particular, the MAW is placed above the Levantine Intermediate Water (LIW), i.e. the intermediate layer of the Mediterranean basin, and includes the euphotic zone of the water column populated by picophytoplankton groups. Moreover, the surface circulation pattern allowed to single out the presence of two main branches of the MAW which cross the Cape Passero-Misurata transect at the water surface: (i) Atlantic Ionian Stream (AIS) localized between the Sicilian coast and Malta; (ii) Atlantic Libyan Current placed close to the Libyan coast. As a consequence, the horizontal velocities take on the same direction of the transect between the Medina Sill and the Libyan coast, while assume a direction non-parallel to the transect close to Cape Passero and Misurata (see Fig. 1 in Supplementary Figures).

The vertical profiles of fluorescence along the whole transect, between the surface and 200 m of depth, indicate that the total *chlorophyll a* concentration takes on the highest values close to the Sicilian coast, due to the strong influence of the thermohaline front present over the Sicilian - Maltese shelf [1, 2]. In the rest of the section, the deep chlorophyll maximum (DCM) is less pronounced and is located between 100 and 150 m of depth. Specifically, the total *chl a* concentration ranges between 0.095 and 0.235 μg *chl a* l^{-1} along the whole transect. Moreover, differences among the twelve hydrological stations are observed in the

shape and width of the DCM.

The chemical analysis performed on the bottle samples confirms that the Mediterranean Sea is a phosphorus limited basin due to the high N:P ratio measured along the water column in all sites investigated [1, 3]. Specifically, the phosphate concentration takes on very low values due to the absorption of Saharan dust, while the higher concentration of nitrate, nitrite and ammonium are strictly connected to: (i) the excess of nitrogen fixation in the Mediterranean basin, (ii) the nutrient flow coming from the coasts.

Phytoplankton properties

In this work, the contribution of each picophytoplankton group to the total amount of chlorophyll is based on the experimental estimation of cellular *chlorophyll a* content obtained by performing the high-performance liquid chromatography (HPLC) analysis on the seawater samples collected between Cape Passero and Malta during a previous oceanographic survey [4, 5].

According to the study carried out in the Mediterranean Sea by other authors [4–7], the phytoplankton community can be divided into three main size fractions: pico- ($< 3\mu m$), nano- ($3 - 20\mu m$) and micro-phytoplankton ($> 20\mu m$). Specifically, in the marine ecosystem studied (Strait of Sicily), the analysis is focused on the picophytoplankton fraction, which accounts, on average, about for 80% of the total *chl a* and *Dvchl a* [4, 5, 8–10], and consists of two main domains: picoprokaryotes and picoeukaryotes. The former is composed of two genera of cyanobacteria, i.e. Synechococcus and Prochlorococcus. The latter is dominated by Prymnesiophytes, Pelagophytes and green algae. On the other hand, the nano- and micro-phytoplankton fraction accounts for about 20% of the total *chl a* and *Dvchl a* on average, and is mainly represented by the eukaryotes domain, i.e. Prymnesiophytes, Pelagophytes, Dinophytes and diatoms. This fraction is poorly present in DCM, and is almost uniformly distributed along the water column.

In this study, we analyze the behaviour of four picophytoplankton populations, i.e. Synechococcus, Prochlorococcus (HL-ecotype), Prochlorococcus (HL-ecotype) and the whole picoeukaryotes domain, which are located at different depths along the water column. Field observations indeed indicate a prevalence of Synechococcus close to the water surface, while Prochlorococcus and picoeukaryotes dominate the intermediate deeper layers [4, 5].

Synechococcus is mostly present close to the coasts, where its cell concentration reaches

usually the maximum value. Moreover, the analyses carried out along the water column showed the presence of nine different phylogenetic groups (clades) of Synechococcus in the Mediterranean Sea, most of which are observed in the Strait of Sicily [9].

The Prochlorococcus concentration is characterized by a bimodal distribution in the intermediate layers of the water column [5, 9], indicating the coexistence of two ecotypes of this genus: high light-adapted (HL-) ecotype and low light-adapted (LL-) ecotype. The former ecotype is localized in the upper part of the MAW between the surface water and 90 m of depth. The latter is mostly present at depths greater than 50 m [5, 8, 9]. Moreover, recent studies showed that the Prochlorococcus HL-ecotype prevails close to the Sicilian coast [4, 5, 9], while Prochlorococcus LL-ecotype dominates the marine ecosystem between the Sicilian - Maltese shelf and the Libyan coast [8, 9]. However, the biological features of both Prochlorococcus ecotypes can change along the Cape Passero - Misurata transect. Indeed, the phylogenetic analyses performed in the Strait of Sicily indicate the presence of two different clades of Prochlorococcus HL-ecotype in the upper layer of the water column, while three different clades of Prochlorococcus LL-ecotype were detected in deeper layers, contemporaneously. It is worth to recall that heterogenous composition is also a feature of the picoeukaryotes domain. Indeed, Brunet et al. found that Prymnesiophytes are more abundant in shallower layers of MAW, while Pelagophytes prevail in deeper layers [4, 5]. However, unlike other marine ecosystems, no previous studies confirmed a clear segregation of groups belonging to the picoeukaryotes domain in the transect investigated.

In our study, the prevalence of different clades, ecotypes and/or groups inside the four picophytoplankton populations, whose dynamics is modeled in this work, is taken into account for each hydrological station of the Cape Passero - Misurata transect. In particular, we use different settings for some biological parameters, such as the half-saturation constants for light intensity and nutrient concentration, in order to consider the phylogenetic modifications among the marine stations investigated.

The analysis of seawater samples shows that Synechococcus contributes on average to more than 20% of the total chlorophyll concentration in the Strait of Sicily [4, 5]. In particular, the average concentration of Synechococcus is 5.8×10^3 cell ml^{-1}, while its concentration peak (1.05×10^4 cell ml^{-1}) is localized at 50 m of depth. The *chl a* cellular content of Synechococcus has never been estimated in the Strait of Sicily. Therefore, assuming the oligotrophic conditions, we chose to use the content measured by Vaulet and Courties in the English Channel waters, whose value was fixed equal to 1.18 fg *chl a* cell^{-1} [11].

In the Strait of Sicily, Prochlorococcus and picoeukaryotes contribute equally to the primary

production in terms of *chl a* and *Dvchl a* concentrations in intermediate and deeper layers of the MAW. However, picophytoplankton is numerically dominated by Prochlorococcus with an average concentration of 5.2×10^4 cell ml^{-1}. Specifically, this group is mainly localized in DCM, where can reach the mean value of 12.5×10^4 cell ml^{-1}. The marker of Prochlorococcus is *divinil chlorophyll a*, whose molecular structure is very similar to that of *chlorophyll a*. The *Dvchl a* cellular content of total Prochlorococcus ranges between 0.25 and 2.20 fg *Dvchl a* cell^{-1} along the water column, with a mean value exponentially increasing with the depth [5].

The analysis performed on the seawaters shows that the average picoeukaryotes concentration in the DCM is 0.6×10^3 cell ml^{-1} [4, 5, 7], while the mean value of *chl a* cell^{-1} ranges between 10 and 660 fg *chl a* cell^{-1} along the water column, with a significant exponential increase with the depth [5]. Therefore, the concentration of *chl a* per picoeukaryotes cell changes within the MAW, assuming significantly higher values in the DCM respect to the upper layer [12, 13].

On the basis of these findings, we convert the picophytoplankton abundances into chlorophyll concentration by using the two conversion curves obtained by Brunet et al. [5] for Prochlorococcus and picoeukaryotes (see Fig. 2 in Supplementary Figures).

Setting of parameters

In this work, we reproduce the two-dimensional distribution of the total chlorophyll concentration experimentally observed in the Strait of Sicily, by setting the values of the environmental and biological parameters so that the monostability condition in the marine ecosystem is obtained. In particular, initially the environmental parameters are estimated by the experimental data collected along the Cape Passero - Misurata transect. Afterwards, on the basis of the environmental conditions observed along the water column, the biological parameters are fixed in such a way to guarantee the coexistence of all four planktonic populations [14–16] in the intermediate and deeper layers. By this way, we mimic the presence of a deep chlorophyll maximum (DCM) in all hydrological stations, although the peak of abundance for each group is localized at a different depth [14–18].

In accordance with the field observations, the hydrodynamical variables are fixed in the model at constant values during the whole sampling period investigated (15-30 July 2008), even if they can change during the year. Since the only field data available in this study

were those acquired during the MedSudMed-08 oceanographic survey (summer season 2008), we did not have any data to reproduce the effects of seasonal changes in hydrological variables during the whole period investigated by the model (approximately two years and three months). However, in previous works [16, 19] it has been shown that, in Mediterranean Sea, the seasonal changes of environmental parameters do not modify significantly the steady spatial distributions of phytoplankton abundance obtained in summer season, since the hydrological variables remain almost constant between late spring and late autumn. For these reasons, the stationarity assumption can be considered valid, even though the steady solution is reached after a very long integration time and the seasonal changes of environmental variables are not taken into account in the model (see main article).

The numerical values assigned to biological and environmental parameters are shown in Table I of Supplementary Tables.

In our model, we introduce some environmental parameters acquired directly in the Strait of Sicily during the summer season. Specifically, as a preliminary step, we assume that the horizontal velocity component, v_h, takes on the same direction of the meridional geostrophic velocity, v, measured in the sampling sites. This choice is correct since the Cape Passero - Misurata transect can be considered almost parallel to the Earth meridian at $15^o 10'$ of east longitude. The spatial distribution of the horizontal velocity component, $v_h(x, z)$, is therefore reproduced by interpolating the vertical profiles of the meridional geostrophic velocity, $v(z)$, collected in each hydrological station, and is inserted in Eqs. (1), (2) and (5) of the model (see main article).

In this work, the vertical turbulent diffusivity is reproduced according to the method by Pacanowski and Philander [20, 21], by using the experimental profiles of geostrophic velocity components and density collected along the Cape Passero - Misurata transect. In particular, to obtain the vertical turbulent diffusivity as a function of the depth, $D_v(z)$, in each sampling site we use the following expression based on the empirical studies [20, 22, 23]:

$$D_v(z) = \frac{\nu_0}{(1 + \alpha \cdot Ri(z))^n} + \nu_b, \tag{1}$$

where $\nu_0 = 36m^2/h$, $\alpha = 5.0$, and $n = 1.0$ are adjustable parameters chosen for lower intensities of wind stress, $\nu_b = 0.36m^2/h$ is the background dissipation parameter, $Ri(z)$ is the gradient Richardson number, depending on the depth, given by

$$Ri(z) = \frac{N^2(z)}{shear^2(z)} \tag{2}$$

Here, the buoyancy frequency $N(z)$ is calculated directly by the vertical profile of water density as follows

$$N^2(z) = (g/\rho_w) \cdot \frac{\partial \rho(z)}{\partial z}, \tag{3}$$

where g is the gravity acceleration, ρ_w is the density of the sea water, and $\frac{\partial \rho(z)}{\partial z}$ is the vertical density gradient. On the other hand, the shear of the mean currents, $shear(z)$, depends on the vertical profiles of the horizontal velocity components according to the following expression [20, 21]

$$shear^2(z) = \left(\frac{\partial u(z)}{\partial z}\right)^2 + \left(\frac{\partial v(z)}{\partial z}\right)^2, \tag{4}$$

where $u(z)$ and $v(z)$ are the zonal and the meridional geostrophic current components measured along the water column in each hydrological station of the Cape Passero - Misurata transect. By this way, we get the two-dimensional distribution of the vertical turbulent diffusivity, $D_v(x, z)$, by interpolating the theoretical profiles obtained for the 12 hydrological stations.

Unlike the other environmental parameters, the horizontal turbulent diffusivity, $D_h(x)$, can not be calculated by using the experimental data collected in the Strait of Sicily. Therefore, the values of this parameter are set in agreement with the theoretical findings obtained in previous works [20, 24, 25]. In particular, in our model the horizontal turbulent diffusivity decreases from the maximum value, $D_h(x) = 36.0\ km^2/h$, (station $M1$, close to the Sicilian coast) to the minimum value, $D_h(x) = 7.2\ km^2/h$, (station $M4$, at the beginning of the Medina Sill). In the other hydrological stations the horizontal turbulent diffusivity is independent of the position x along the transect and is set to $7.2\ km^2/h$, according to theoretical results obtained by Katz et al. for deeper waters [20, 24].

The one-dimensional behaviour of the incident light intensity at the water surface ($I_{in}(x)$) is estimated by using the remote sensing data (see the NASA web site http://eosweb.larc.nasa.gov/sse/RETScreen/) acquired for each station of the transect, while the two-dimensional distribution of the phosphate concentration at the initial time ($t = 0$) is set on the basis of the analysis performed on the bottle samples collected at different depths in the hydrological stations. By this way, we also obtain the phosphate concentration at the boundaries of the two-dimensional domain, $R_{in}(x, z)$, necessary to solve the equations of the model.

The biological parameters are set to values typical of the four phytoplankton populations studied, according to previous theoretical and experimental results [4, 5, 11, 12, 18, 26–35]. In particular, the maximum specific growth rates are fixed in accordance with experimental results given in Refs. [28–30], while the specific loss rates are estimated on the basis of

experimental findings given in Refs. [28, 30–32]. On the other hand, the swimming velocity and nutrient recycling coefficients are chosen in agreement with the theoretical results obtained by other authors [27, 31]. Specifically, the magnitudes of swimming velocities of the four planktonic populations are set to the values obtained by Raven [27], while nutrient recycling coefficients are calculated by considering the assimilation efficiencies estimated by Thingstad [31].

In previous works [14–16], as well as in our preliminary analysis, the half-saturation constants of the picophytoplankton groups are fixed to the average values observed experimentally (see parameters of the full model in Table I). In particular, the half-saturation constants K_{I_i} are fixed at low values for those populations, such as picoeukaryotes and Prochlorococcus LL, which are better adapted to low light intensities. At the same time, the half-saturation constants K_{R_i} are set at low values for those populations, such as Synechococcus and Prochlorococcus HL, which are better adapted to low nutrient concentrations. Therefore, the abundance peaks of picoeukaryotes and Prochlorococcus LL are localized in the deeper layers of the MAW, while those of the Synechococcus and Prochlorococcus HL are placed in the intermediate layers.

Conversely, on the basis of the field observations, the core of this study exploits half-saturation constants set to different values in the twelve hydrological stations investigated (see parameters of the reduced model in Table I). Indeed we recall that in the Strait of Sicily the picoeukaryotes domain includes several groups [4, 5], while Synechococcus, Prochlorococcus HL and Prochlorococcus LL are characterized by the presence of different clades [8, 9]. For each phytoplankton population this biodiversity is a marker of the skills of adaptation at the different environmental conditions observed within the 2D domain of the marine ecosystem. As a consequence, depending on the marine site analyzed, different clades, ecotypes and/or groups prevail inside each population, and the half-saturation constants have to change accordingly [36]. Thus, the half-saturation constants, K_{R_i} and K_{I_i}, for the four populations are set so that the production layers and the peaks of phytoplankton abundance are placed at depths compatible with those observed by Brunet et al. [4, 5] in the Strait of Sicily. In particular, according to Ryabov et al., for the i-th population and for each hydrological station, we consider the following expressions

$$K_{R_i} = \frac{(r_i - m_i)}{m_i} R_i^* \tag{5}$$

$$K_{I_i} = \frac{(r_i - m_i)}{m_i} I_i^*, \tag{6}$$

where R_i^* and I_i^* are the critical values, defined as the values of the resource availability at the boundaries of the production layer, r_i is the maximum specific growth rate, and m_i is the specific loss rate [15, 37]. As a preliminary step, we calculate the values of half-saturation constants by using in Eqs. (5), (6) the critical values of nutrient concentration and light intensity obtained from the field data. The values of half-saturation constants estimated in this way are successively adjusted by using again Eqs. (5), (6), in which new values for R_i^* and I_i^*, calculated from the model, are used instead of those obtained from the field data. By applying iteratively this procedure, the values of half-saturation constants are refined in such a way to optimize the chlorophyll distributions obtained from the model compared with the experimental profiles.

Conversely, the nutrient contents of the planktonic populations, $1/Y_i$, are fixed to the same values for all hydrological stations (see Table I). Specifically, the values of these parameters are estimated for Synechococcus and picoeukaryotes according to the experimental findings obtained in previous works [34, 35], while no data are available for any Prochlorococcus ecotype (neither HL, nor LL). Therefore, in order to get phytoplankton abundances in accordance with the experimental results, we fix the nutrient content of the Prochlorococcus (both ecotypes) in such a way to respect the ratios of the average concentrations of the planktonic groups experimentally observed in the Strait of Sicily [4, 5].

Finally, the *chl a*-normalized average absorption coefficients are calculated on the basis of the light absorption spectra obtained for phytoplankton cultures by Hickman et al. [16, 18]. In general, the values estimated are in agreement with the absorption coefficients measured by Brunet et al. in the coastal zones of the Mediterranean Sea [12, 13].

Statistical analysis

Since some parameters (seven half-saturation constants) are freely estimated in the model, it is worth to investigate the real goodness of the fit obtained by performing a comparison, based on the χ^2 test, between the reduced model and the full model [39]. Specifically, in order to establish which of them reproduces better the field data, we applied for both models the Akaike Information Criterion (AIC) [38, 39] defined as follows

$$AIC(H) = \chi^2_{df} - df, \tag{7}$$

where χ^2_{df} denotes a chi-squared with a number of degrees of freedom, df, equal to the

number of independent constraints of the model, H. The minimum value of AIC indicates the model with the best fit.

In Table II, we show the AIC statistics calculated for each hydrological station and the whole transect. Here, the results indicate that the best AIC in the most sites (eleven hydrological stations over twelve) is obtained by the full model, while the χ^2 test show that the best fit is usually observed by using the reduced model. However, for the whole transect we observe that the best AIC statistics is obtained by using the reduced model, in accordance with the result of the χ^2 test.

Therefore, on this basis the reduced model seems to be the best tool to reproduce the overall two-dimensional distribution of chlorophyll concentration. On the other hand, the full model is able to well reproduce the experimental chlorophyll profiles in the most sites of the transect.

This conclusion can be further checked by using an other statistical tool, which defines a magnitude difference between the chi-square for the reduced model and the chi-square for the full model. In particular, we estimate this magnitude difference by using the Cohen's effect size measure [39]

$$w = (\triangle\chi^2_{df}/(N \cdot \triangle df))^{0.5}, \tag{8}$$

where N is the sample size, equal for both models, $\triangle\chi^2_{df}$ is the difference in chi-square and $\triangle df$ the difference in degrees of freedom between the two models.

According to the Cohen's suggested standard for a small effect ($w \leq 0.1$) [39], the value obtained for the index w indicates that using the reduced model affects weakly (see Table II of Supplementary Tables) the result of the χ^2 test in the whole transect ($w = 0.08$). Specifically, according to the values of w, the decrease of χ^2 is negligible in the sites from $M4$ to $M12$, while it is more significative, although still small, in the three hydrological stations localized close to the Sicilian coast. On the whole, the reduced model therefore allows to improve the results of the χ^2 test compared to the full model, even if this improvement is not actually very significative.

SUPPLEMENTARY REFERENCES

[1] Placenti, F. et al. Water masses and nutrient distribution in the Gulf of Syrte and between Sicily and Libya. *J. Marine Syst.* **121–122**, 36–46 (2013).

[2] Bonanno, A. et al. Variability of water mass properties in the Strait of Sicily in summer period of 1998–2013. *Ocean Sci.* **10**, 759–770 (2014).

[3] Ribera d'Alcalà, M., Civitarese, G., Conversano, F., Lavezza, R. Nutrient ratios and fluxes hint at overlooked processes in the Mediterranean Sea. *J. Geophys. Res.* **108(C9)**, 8106 (2003).

[4] Brunet, C., Casotti, R., Vantrepotte, V., Corato, F., Conversano, F. Picophytoplankton diversity and photoacclimation in the Strait of Sicily (Mediterranean Sea) in summer. I. Mesoscale variations. *Aquat. Microb. Ecol.* **44**, 127–141 (2006).

[5] Brunet, C., Casotti, R., Vantrepotte, V., Conversano, F. Vertical variability and diel dynamics of picophytoplankton in the Strait of Sicily, Mediterranean Sea, in summer. *Mar. Ecol. Prog. Ser.* **346**, 15–26 (2007).

[6] Casotti, R., Brunet, C., Aronne, B., Ribera d'Alcalà, M. Mesoscale features of phytoplankton and planktonic bacteria in a coastal area as induced by external water masses. *Mar. Ecol. Prog. Ser.* **195**, 15–27 (2000).

[7] Casotti, R. et al. Composition and dynamics of the phytoplankton of the Ionian Sea (Eastern Mediterranean). *J. Geophys. Res.* **108(C9)**, 8116 (2003).

[8] Garczarek, L. et al. High vertical and low horizontal diversity of *Prochlorococcus* ecotypes in the Mediterranean Sea in summer. *FEMS Microbiol Ecol.* **60**, 189–206 (2007).

[9] Mella-Flores, D. et al. Is the distribution of Prochlorococcus and Synechococcus ecotypes in the Mediterranean Sea affected by global warming? *Biogeosciences* **8(9)**, 2785–2804 (2011).

[10] La Ferla, R. et al. Vertical distribution of the prokaryotic cell size in the Mediterranean Sea. *Helgol. Mar. Res.* **66(4)**, 635–650 (2012).

[11] Morel, A., Ahn, Y.H., Partensky, F., Vaulot, D., Claustre, H. *Prochlorococcus* and *Synechococcus*: A comparative study of their optical properties in relation to their size and pigmentation. *J. Mar. Res.* **51**, 617–649 (1993).

[12] Brunet, C., Casotti, R., Aronne, B., Vantrepotte, V. Measured photophysiological parameters used as tools to estimate vertical water movements in the coastal Mediterranean. *J. Plankton*

Res. **25**, 1413–1425 (2003).

[13] Brunet, C., Casotti, R., Vantrepotte, V. Phytoplankton diel and vertical variability in photobiological responses at a coastal station in the Mediterranean Sea. *J. Plankton Res.* **30**, 645–654 (2008).

[14] Huisman, J., Thi, N.N.P., Karl, D.M., Sommeijer, B. Reduced mixing generates oscillations and chaos in the oceanic deep chlorophyll maximum. *Nature* **439**, 322–325 (2006).

[15] Ryabov, A.B., Rudolf, L., Blasius, B. Vertical distribution and composition of phytoplankton under the influence of an upper mixed layer. *J. Theor. Biol.* **263**, 120–133 (2010).

[16] Valenti, D., Denaro, G., Spagnolo, B., Conversano, F., Brunet, C. How diffusivity, thermocline and incident light intensity modulate the dynamics of deep chlorphyll maximum in Tyrrhenian Sea. *PLoS ONE* **10(1)**, e0115468 (2015).

[17] Klausmeier, C.A., Litchman, E. Algal games: the vertical distribution of phytoplankton in poorly mixed water columns. *Limnol. Oceanogr.* **46**, 1998–2007 (2001).

[18] Hickman, A., Dutkiewicz, S., Williams, R., Follows, M. Modelling the effects of chromatic adaptation on phytoplankton community structure in the oligotrophic ocean. *Mar. Ecol. Prog. Ser.* **406**, 1–17 (2010).

[19] Valenti, D. et al. Stochastic models for phytoplankton dynamics in Mediterranean Sea. *Ecol. Complex.* **27**, 84–103 (2016).

[20] Pacanowski, R.C., Philander, S.G.H. Parameterization of Vertical Mixing in Numerical Models of Tropical Oceans. *J. Phys. Oceanogr.* **11**, 1443–1451 (1981).

[21] Peters, H., Gregg, M.C., Toole, J.M. On the parameterization of Equatorial Turbulence. *J. Geophys. Res.* **93**, 1199–1218 (1988).

[22] Robinson, A.R. An investigation into the wind as the cause of the Equatorial Undercurrent. *J. Mar. Res.* **24**, 179–204 (1966).

[23] Jones, J.H. Vertical mixing in the Equatorial Undercurrent. *J. Phys. Oceanogr.* **3**, 286–296 (1973).

[24] Katz, E.J., Bruce, J.G., Petrie, B.D. Salt and mass flux in the Atlantic Equatorial Undercurrent. *Deep-Sea Res.* **26**, 139–160 1979.

[25] Massel, S.R. Fluid Mechanics for Marine Ecologists. (Springer-Verlag, Berlin Heidelberg, 1999).

[26] Morel, A. Consequences of a Synechococcus bloom upon the optical properties of oceanic (case 1) waters. *Limnol. Oceanogr.* **42(8)**, 1746–1754 1997.

[27] Raven, J.A., The twelfth tansley lecture. Small is beautiful: the picophytoplankton. *Funct.*

Ecol. **12**, 503–513 (1998).

[28] Raven, J.A., Finkel, Z.V., Irwin, A.J. Picophytoplankton: bottom-up an top-down controls on ecology and evolution. *J. Geophys. Res.* **55**, 209–215 (2005).

[29] Dimier, C., Brunet, C., Geider, R., Raven, J. Growth and photoregulation dynamics of the picoeukaryote *Pelagomonas calceolata* in fluctuating light. *Limnol. Oceanogr.* **54**, 823–836 (2009).

[30] Veldhuis, M.J.W., Timmermans, K.R., Croot, P., Van Der Wagt, B. Picophytoplankton; a comparative study of their biochemical composition and photosynthetic properties. *J. Sea Res.* **53**, 7–24 (2005).

[31] Thingstad, T.F., Sakshaugh, E. Control of phytoplankton growth in nutrient recycling ecosystems. Theory and terminology. *Mar. Ecol. Prog. Ser.* **63**, 261–272 (1990).

[32] Quevedo, M., Anadón, R. Protist control of phytoplankton growth in the subtropical northeast Atlantic. *Mar. Ecol. Prog. Ser.* **221**, 20–38 (2001).

[33] Moore, L.R., Goericke, R., Chisholm, S.W. Comparative physiology of synechococcus and prochlorococcus: influence of light and temperature on growth, pigments, fluorescence and absorptive properties. *Mar. Ecol. Prog. Ser.* **116**, 259–275 (1995).

[34] Bertilsson, S., Berglund, O., Karl, D.M., Chisholm, S.W. Elemental composition of marine *Prochlorococcus* and *Synechococcus*: implications for the ecological stoichiometry of the sea. *Limnol. Oceanogr.* **48**, 1721–1731 (2003).

[35] Timmermans, K.R., van der Wagt, B., Veldhuis, M.J.W., Maatman, A., de Baar, H.J.W. Physiological responses of three species of marine pico-phytoplankton to ammonium, phospahte, iron and light limitation. *J. Sea Res.* **53**, 109–120 (2005).

[36] Moon-Van Der Staay, S.Y., De Wachter, R., Vaulot, D. Oceanic 18S rDNA sequences from picoplankton reveal unsuspected eukaryotic diversity. *Nature* **409**, 607–610 (2001).

[37] Ryabov, A. Phytoplankton competition in deep biomass maximum. *Theor. Ecol.* **5**, 373–385 (2012).

[38] Akaike, H. Factor analysis and AIC. *Psychometrika* **52**, 317–332 (1987).

[39] Newsom, J.T. *Longitudinal Structural Equation Modeling: A Comprehensive Introduction* (Routledge, New York, NY, 2015).

SUPPLEMENTARY TABLES

Symbol	Interpretation	Units	Reduced Model	Full Model
a_{bg}	Background turbidity	m^{-1}	0.060	0.060
a_1	Average absorption coefficient of Synechococcus	m^2 mg chl-a $^{-1}$	0.025	0.025
a_2	Average absorption coefficient of Prochlorococcus HL	m^2 mg chl-a $^{-1}$	0.016	0.016
a_3	Average absorption coefficient of picoeukaryotes	m^2 mg chl-a $^{-1}$	0.012	0.012
a_4	Average absorption coefficient of Prochlorococcus LL	m^2 mg chl-a $^{-1}$	0.027	0.027
a_6	Average absorption coefficient of phytoplankton $> 3\mu m$	m^2 mg chl-a $^{-1}$	0.020	0.020
r_1	Maximum specific growth rate of Synechococcus	h^{-1}	0.058	0.058
r_2	Maximum specific growth rate of Prochlorococcus HL	h^{-1}	0.088	0.088
r_3	Maximum specific growth rate of picoeukaryotes	h^{-1}	0.096	0.096
r_4	Maximum specific growth rate of Prochlorococcus LL	h^{-1}	0.031	0.031
K_{I_1}	Half-saturation constant of light-limited growth of Synechococcus (as a function of distance)	μmol photons m^{-2} s^{-1}	12.50 – 130.00	130.00
K_{I_2}	Half-saturation constant of light-limited growth of Prochlorococcus HL (as a function of distance)	μmol photons m^{-2} s^{-1}	24.50 – 80.00	80.00
K_{I_3}	Half-saturation constant of light-limited growth of picoeukaryotes (as a function of distance)	μmol photons m^{-2} s^{-1}	16.50 – 67.50	67.50
K_{I_4}	Half-saturation constant of light-limited growth of Prochlorococcus LL (as a function of distance)	μmol photons m^{-2} s^{-1}	0.01 – 24.50	1.00
K_{R_1}	Half-saturation constant of nutrient-limited growth of Synechococcus	mmol phosphorus m^{-3}	0.000	0.000
K_{R_2}	Half-saturation constant of nutrient-limited growth of Prochlorococcus HL (as a function of distance)	mmol phosphorus m^{-3}	0.000 – 0.365	0.060
K_{R_3}	Half-saturation constant of nutrient-limited growth of picoeukaryotes (as a function of distance)	mmol phosphorus m^{-3}	0.000 – 1.000	0.098
K_{R_4}	Half-saturation constant of nutrient-limited growth of Prochlorococcus LL (as a function of distance)	mmol phosphorus m^{-3}	0.000 – 0.095	0.040
m_1	Specific loss rate of Synechococcus	h^{-1}	0.014	0.014
$m_2 = m_4$	Specific loss rate of Prochlorococcus	h^{-1}	0.011	0.011
m_3	Specific loss rate of picoeukaryotes	h^{-1}	0.010	0.010
$1/Y_1$	Nutrient content of Synechococcus	mmol phosphorus $cell^{-1}$	2.0×10^{-12}	2.0×10^{-12}
$1/Y_2 = 1/Y_4$	Nutrient content of Prochlorococcus	mmol phosphorus $cell^{-1}$	4.0×10^{-13}	4.0×10^{-13}
$1/Y_3$	Nutrient content of picoeukaryotes	mmol phosphorus $cell^{-1}$	2.00×10^{-12}	2.00×10^{-12}
c_1	Chl-a cellular content of Synechococcus	fg chl-a $cell^{-1}$	1.18	1.18
c_3	Chl-a cellular content of picoeukaryotes (as a function of depth)	fg chl-a $cell^{-1}$	10.00 – 660.00	10.00 – 660.00
$c_2 = c_4$	Dvchl-a cellular content of Prochlorococcus (as a function of depth)	fg Dvchl-a $cell^{-1}$	0.25 – 2.20	0.25 – 2.20
ε_1	Nutrient recycling coefficient of Synechococcus	dimensionless	0.51	0.51
$\varepsilon_2 = \varepsilon_4$	Nutrient recycling coefficient of Prochlorococcus	dimensionless	0.52	0.52
ε_3	Nutrient recycling coefficient of picoeukaryotes	dimensionless	0.52	0.52
v_1^s	Magnitude of swimming velocity of Synechococcus	m h^{-1}	0.000088	0.000088
$v_2^s = v_4^s$	Magnitude of swimming velocity of Prochlorococcus	m h^{-1}	0.000039	0.000039
v_3^s	Magnitude of swimming velocity of picoeukaryotes	m h^{-1}	0.000098	0.000098
D_V	Vertical turbulent diffusivity (as a function of depth and distance)	m^2 h^{-1}	0.360 – 22.08	0.360 – 22.08
D_H	Horizontal turbulent diffusivity (as a function of distance)	km^2 h^{-1}	7.2 – 36.0	7.2 – 36.0
z_b	Depth of the water column (as a function of distance)	m	50 – 200	50 – 200
R_{in}	Nutrient concentration at the domain boundaries	mmol phosphorus m^{-3}	0.010 – 0.110	0.010 – 0.110

TABLE I: Parameters used for reduced and full model. The values of the biological and environmental parameters are those typical of four picophytoplankton populations that coexist in the Strait of Sicily.

Station	χ^2_{red}	$\tilde{\chi}^2_{red}$	χ^2_{ful}	$\tilde{\chi}^2_{ful}$	AIC_{red}	AIC_{ful}	w
$M1$	1.27	0.0508	6.55	0.2620	−68.73	−77.45	0.17
$M2$	1.63	0.0271	23.44	0.3904	−68.37	−60.58	0.23
$M3$	0.64	0.0112	11.57	0.2029	−69.36	−72.43	0.17
$M4$	1.19	0.0145	1.96	0.0239	−68.81	−82.04	0.04
$M5$	1.19	0.0120	2.71	0.0274	−68.81	−81.29	0.05
$M6$	0.43	0.0059	1.06	0.0145	−69.57	−82.94	0.04
$M7$	1.00	0.0100	0.84	0.0084	−69.00	−83.16	0.02
$M8$	0.72	0.0072	0.83	0.0083	−69.28	−83.17	0.01
$M9$	1.12	0.0113	5.07	0.0512	−68.88	−78.93	0.08
$M10$	1.52	0.0152	0.47	0.0047	−68.48	−83.53	0.04
$M11$	1.99	0.0199	1.84	0.0184	−68.01	−82.16	0.01
$M12$	0.11	0.0021	0.39	0.0079	−69.89	−83.61	0.03
$Transect$	12.80	0.0136	56.70	0.0600	−57.20	−27.30	0.08

TABLE II: Results of χ^2 test, reduced chi-square ($\tilde{\chi}^2$) test, Akaike Information Criterion (AIC), and Cohen's effect size measure (w) for the twelve hydrological stations and the whole transect investigated. All statistical tests are carried out for the reduced model (χ^2_{red}, $\tilde{\chi}^2_{red}$ and AIC_{red}) and the full model (χ^2_{ful}, $\tilde{\chi}^2_{ful}$ and AIC_{ful}). The Cohen's effect size measure (w) is calculated on the basis of χ^2 tests, according to Eq.(8). The number of samples, used for the test and distanced of 2 m, depends on the depth of the MAW in each station.

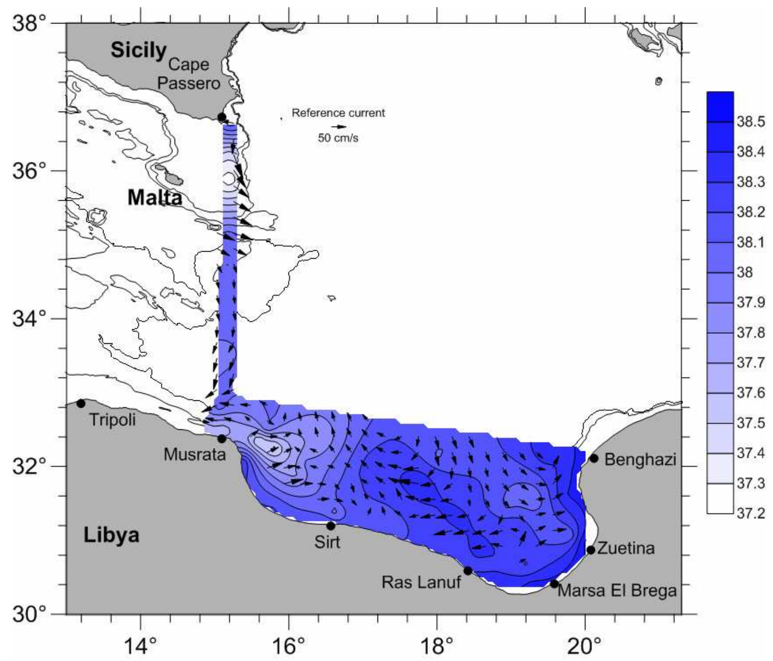

FIG. 1: Horizontal distribution of interpolated LADCP current velocities and minimum of salinity in the upper layer (10 - 80 m). Map was obtained by Surfer [12] from Golden Software, LLC (www.goldensoftware.com).

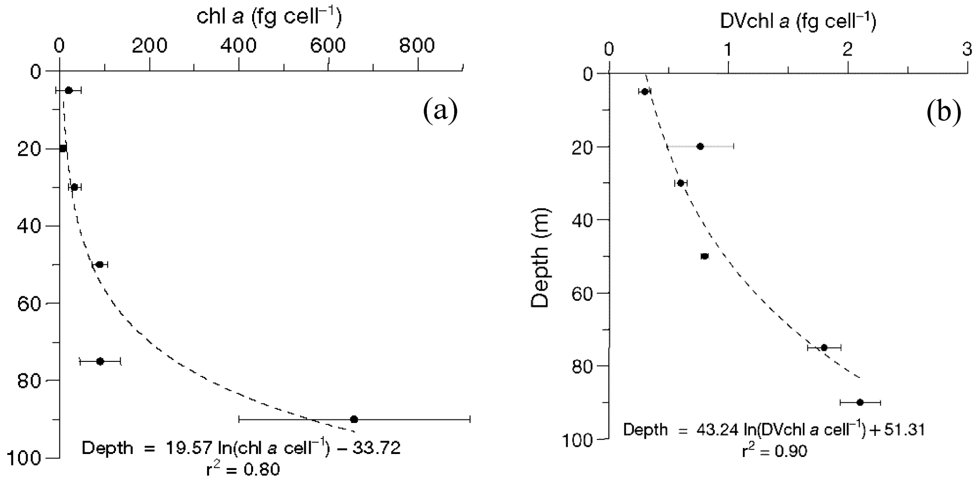

FIG. 2: Mean vertical profile of *chl a* per picoeukaryote cell (panel a) and *Dvchl a* per Prochlorococcus cell (panel b). Error bars are Standard Deviation. Equation and r^2 for the fit are reported on the plots. (Courtesy of Brunet et al., 2007 (Ref. [5])).
